# Identifying congestion phenotypes using unsupervised machine learning in acute heart failure

**DOI:** 10.1093/ehjdh/ztaf065

**Published:** 2025-07-15

**Authors:** Tripti Rastogi, Olivier Hutin, Jozine M ter Maaten, Guillaume Baudry, Luca Monzo, Emmanuel Bresso, Kevin Duarte, Jasper Tromp, Adriaan A Voors, Nicolas Girerd

**Affiliations:** Université de Lorraine, Inserm, DCAC, Centre D’Investigation Clinique—Plurithématique 14-33, CHRU-Nancy, F-CRIN iNI-CRCT (Cardiovasculaire and Renal Clinical Trialists), 4, rue du Morvan, 54500 Vandœuvre-Lès-Nancy, France; Université de Lorraine, Inserm, DCAC, Centre D’Investigation Clinique—Plurithématique 14-33, CHRU-Nancy, F-CRIN iNI-CRCT (Cardiovasculaire and Renal Clinical Trialists), 4, rue du Morvan, 54500 Vandœuvre-Lès-Nancy, France; Department of Cardiology, University of Groningen, University Medical Center Groningen, Groningen, The Netherlands; Université de Lorraine, Inserm, DCAC, Centre D’Investigation Clinique—Plurithématique 14-33, CHRU-Nancy, F-CRIN iNI-CRCT (Cardiovasculaire and Renal Clinical Trialists), 4, rue du Morvan, 54500 Vandœuvre-Lès-Nancy, France; Université de Lorraine, Inserm, DCAC, Centre D’Investigation Clinique—Plurithématique 14-33, CHRU-Nancy, F-CRIN iNI-CRCT (Cardiovasculaire and Renal Clinical Trialists), 4, rue du Morvan, 54500 Vandœuvre-Lès-Nancy, France; Université de Lorraine, Inserm, DCAC, Centre D’Investigation Clinique—Plurithématique 14-33, CHRU-Nancy, F-CRIN iNI-CRCT (Cardiovasculaire and Renal Clinical Trialists), 4, rue du Morvan, 54500 Vandœuvre-Lès-Nancy, France; Université de Lorraine, Inserm, DCAC, Centre D’Investigation Clinique—Plurithématique 14-33, CHRU-Nancy, F-CRIN iNI-CRCT (Cardiovasculaire and Renal Clinical Trialists), 4, rue du Morvan, 54500 Vandœuvre-Lès-Nancy, France; Department of Cardiology, University of Groningen, University Medical Center Groningen, Groningen, The Netherlands; Saw Swee Hock School of Public Health and National University of Singapore and National University Health System, 12 Science Drive 2, #10-01, Singapore; Duke-NUS Medical School Singapore, 8 College Rd, Singapore; Department of Cardiology, University of Groningen, University Medical Center Groningen, Groningen, The Netherlands; Université de Lorraine, Inserm, DCAC, Centre D’Investigation Clinique—Plurithématique 14-33, CHRU-Nancy, F-CRIN iNI-CRCT (Cardiovasculaire and Renal Clinical Trialists), 4, rue du Morvan, 54500 Vandœuvre-Lès-Nancy, France

**Keywords:** Heart failure phenotypes, Congestion, Protein biomarkers, Random forest, Unsupervised clustering

## Abstract

**Aims:**

Data-driven clustering techniques may improve heart failure (HF) categorisation and provide prognostic insights. The present study aimed to elucidate the underlying pathophysiology of acute HF phenotypes based on pulmonary and systemic congestion at both the tissue (PTC, pulmonary tissue congestion; STC, systemic tissue congestion) and intravascular (PIVC, pulmonary intravascular congestion; SIVC, systemic intravascular congestion) level and to assess the association of identified phenotypes with a composite outcome of HF hospitalisation and death.

**Methods and results:**

Nineteen clinical, laboratory, and echocardiographic congestion markers were analyzed using clustering techniques to identify phenotypes in patients with worsening HF in the Nancy-HF cohort (*n* = 741), followed by validation of the clustering model in the BIOSTAT-CHF cohort (*n* = 4254). Network analysis was conducted using 363 proteins to identify underlying biological pathways. Five congestion phenotypes were identified: (1) PTC-dilated left ventricle (LV), (2) PTC-HFpEF, (3) PTC, STC-atrial fibrillation (AF), (4) PIVC-dilated left atrium (LA) and LV and (5) Global congestion. Compared with the ‘PTC-dilated LV’ phenotype, the risk of composite outcome was higher in ‘PTC, STC-AF’ and ‘Global’ congestion phenotypes [adjusted HR: 1.74 (1.13–2.67) and 2.41 (1.60–3.63), respectively]. In BIOSTAT-CHF, ‘Global’ congestion phenotype was associated with significantly higher risk [HR: 1.64 (1.04–2.58)]. In network analysis, the immune response pathway was linked to all phenotypes. ‘PTC-HFpEF’ was related to lipid, protein and angiotensin metabolism, ‘PTC, STC-AF’ was related to kinase-mediated signalling, extracellular matrix organisation and TNF-regulated cell death, while ‘PIVC-dilated LA & LV’ was related to kinase-mediated signalling and hemostasis.

**Conclusion:**

In worsening HF, clustering techniques identified clinical congestion profiles associated with both long-term clinical risk and differences in biomarkers, suggesting potential different underlying pathophysiologies. These clusters can be applied using the available online model to identify phenotypes as well as associated risks (https://cic-p-nancy.fr/ai-cong-hf/).

## Graphical Abstract

**Graphical Abstract:**
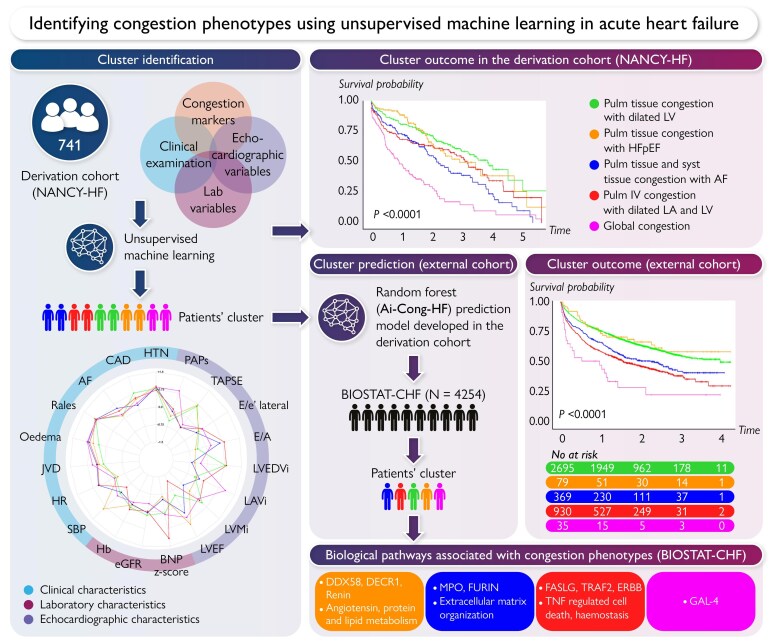

## Introduction

Congestion is a key feature of acute heart failure (AHF) that influences both short- and long-term prognosis.^1^ Congestion may be causally linked with deteriorating heart function by activating inflammatory, neuro-hormonal and oxidative stress pathways.^2–4^ De-congestion is one of the key therapeutic goals in acute and chronic HF.^5^ However, available decongestion therapies remain inadequate.^6^ An optimal combination of ARNi, beta-blockers, mineralocorticoid antagonist and SGLT2i improves prognosis in HF patients.^7,8^ Yet, this combination therapy remains inadequate in a sizeable population, and its implementation is often limited by presence of clinical inertia or comorbidities such as hypotension, electrolyte imbalance, renal dysfunction.^9,10^ Approximately 30–45% of AHF patients are re-admitted or die within 1 year of discharge.^11,12^ Moreover, a large heterogeneity in congestion types, severity and distribution is suggested to be one of the factors behind inadequate treatment response.^2^ Identifying homogenous ‘phenotypes’ amongst the heterogeneous HF population hence appears as a logical first step towards individualisation of therapy from the current ‘one size fits all’ approach. Furthermore, the ESC-HF guidelines 2021 suggest that a better phenotyping of AHF may improve treatment and prognosis.^11^ Further compounding the issue, the pathophysiology of congestion scenarios is yet to be elucidated, likely due to the absence of easily identifiable phenotypes.

Unsupervised machine learning approaches recently identified distinct ‘phenotypes’ of AHF that go beyond the ‘wet/dry’ and/or left ventricular ejection fraction (LVEF)-based classification. These distinct AHF phenotypes differ both in their prognosis and response to available therapies.^13–15^ Clustering approaches can support clinical decision-making regarding treatment individualization through the identification of AHF phenotypes by analyzing a multitude of data points on demographics, comorbidities, echocardiographic findings and/or circulating protein biomarkers. However, studies assessing congestion phenotypes of AHF based on clinical and echocardiographic congestion variables are currently sparse.^14,16^ Given the impact of congestion on patient survival, we hypothesized that congestion markers-based phenotyping may be more propitious in classifying AHF patients and discovering treatment targets.

In the present study, we sought to unveil the complex pathophysiology of congestion phenotypes in patients with AHF by applying a cluster analysis to categorize these phenotypes based on a comprehensive array of variables, including clinical signs, symptoms, blood tests, and echocardiographic data. To validate and broaden our understanding of these phenotypes, the resulting model was applied in a validation cohort and a network analysis was conducted on circulating protein data to explore the biological underpinnings, assessing their prognostic value by examining associations with clinical outcomes.

## Methods

### Derivation cohort

For cluster identification, patients with HF admitted for worsening HF included in the Nancy-HF cohort (*n* = 741) from January 2015 to May 2019 at the Nancy University Hospital were analyzed. Patients were identified using ICD-9 codes related to HF. The details of the study design and population have been published previously.^17^

### Variable selection and identification of clusters using machine learning

Variables typically used for assessing and quantifying congestion in routine clinical practice were considered. In addition, variables potentially impacting congestion assessment were also included for model building. Variables with >30% missing values were discarded from the analysis, leading to removal of the A wave, e′ mean and E/e′ mean variables. However, E/A was retained for analysis despite 41% missing values given its value as an important indicator of diastolic left ventricular dysfunction and, E/e′ lateral was used instead of E/e′ mean.

The variables used in the final clustering model included congestion variables, namely rales, peripheral oedema, jugular venous distension (JVD), haemoglobin, BNP/NT-proBNP *z*-score, left atrial volume index (LAVi), pulmonary artery systolic pressure (PASP), E/e′ lateral and E/A ratio, as well as variables necessary for adequately assessing the prognostic impact of congestion, or the contextual interpretation of congestion, namely hypertension, coronary artery disease (CAD), known history of atrial fibrillations (AF), heart rate, systolic blood pressure (SBP), estimated glomerular filtration rate (eGFR), LVEF, left ventricular mass index (LVMi), left ventricular end-diastolic volume index (LVEDVi), and tricuspid annular plane systolic excursion (TAPSE). Of note, AF is a good example of a variable having a sizeable impact on how echocardiographic variables are interpreted—having AF and other congestion-related variables as input variables in the clustering approach allows more adequate deciphering of the homogeneous groups by considering covariates that modify the interpretation of congestion variables. BNP *z* score was used to harmonize data with NTproBNP data available in the validation cohort.

The clustering analysis was conducted using the VarSelLCM package in R and the optimal number of clusters was identified using the Bayesian Information Criterion. Random Forest (RF) (generating 250 trees), XGBoost, Support Vector Machine and Decision Tree prediction models (7 instances per leaf) with 10-fold cross-validation were subsequently trained in Weka and R software and compared for their performance. Prediction models included age, sex, and BMI, as well as all variables used to identify the aforementioned clusters.

After identifying the clusters, a labelling process was applied to characterize clusters based on congestion phenotype. The labels align with the framework proposed by recent reviews focusing on congestion, which classify patients according to the regional (pulmonary vs. systemic) and compartmental (intravascular vs. tissue) distribution of congestion.^18,19^ The following features were considered:

1. Pulmonary Tissue Congestion (PTC): Characterized by a high frequency of rales.
2. Systemic Tissue Congestion (STC): Defined by a high frequency of peripheral oedema.
3. Systemic Intravascular Congestion (SIVC): Identified by a high frequency of JVD.
4. Pulmonary Intravascular Congestion (PIVC): Associated with elevated BNP levels, high PASP, increased LAVi, and echocardiographic markers such as E/e′ lateral and E/A.

Clusters were labelled as having one or several of these congestion features.

### Validation of the RF model in BIOSTAT-CHF

The RF model derived in the Nancy-HF cohort was validated in the BIOSTAT-CHF cohort (*n* = 4254, pooled data of BIOSTAT-CHF index and BIOSTAT-CHF validation cohorts), a prospective observational cohort that included patients with signs and symptoms of new-onset or worsening HF.^20^ In BIOSTAT-CHF, the LAV was calculated from LA diameter using the linear equation derived in the STANISLAS cohort.^21^ E/e′ lateral, TAPSE and PASP were unavailable and therefore considered missing in the analysis.

### Protein biomarkers in BIOSTAT-CHF

To better identify the pathophysiological background of congestion clusters, 368 protein biomarkers from the OLINK® CVD II, CVD III, Immune Response and Oncology II panels were assessed in BIOSTAT-CHF. Of the 368 proteins assessed in the validation cohort, 5 protein biomarkers (TNF r1, Cstb, Pi3, Klk6, and Azul1) were removed as >10% of protein values were below the limit of detection. A multinomial regression analysis was next conducted to identify proteins significantly associated with each phenotype, with overall *P*-values corrected for false discovery rate (FDR) as described in the statistical analysis section.

### Complex network analysis

The proteins that were uniquely associated with any of the congestion phenotypes were selected for network analysis. Protein annotations were extracted from the FHF graph knowledge box (FHF-GKBox)^22^ from which protein–pathway relationships were extracted from Reactome (version 69).^23^ The FHF-GKBox was searched using query patterns, which are templates predefined in the Cypher Query language, allowing to systematically extract sub-graphs from a Neo4J graph database. Two query patterns were employed, depending on the number of proteins significantly associated with each phenotype: firstly, a biomarker-pathway-biomarker query pattern was defined and used to retrieve pathways linking at least two distinct biomarkers; secondly, a pathway-biomarker pattern was employed to identify all pathways that were significantly over-represented. The resulting sub-graph encompassing all identified pathways and related biomarkers was automatically visualized using the Cytoscape software.^24^

### Outcome in the Nancy-HF and BIOSTAT-CHF cohorts

The outcome was a composite of hospitalisation for HF or death in the derivation cohort and the validation cohort.

#### Statistical analysis

For the descriptive analyses, continuous variables are expressed as mean ± standard deviation (SD) for normally distributed data or as median (Q1-Q3) for skewed data. Categorical variables are expressed as proportions (%). Comparison of baseline characteristics was performed using ANOVA or the Kruskal–Wallis test as required.

Distribution pattern of outcome was examined across all identified phenogroups, stratified by age, sex and LVEF category. Survival probabilities were plotted using Kaplan-Meier curves. The phenotype with the best survival was considered as reference. Cox proportional hazard models for identified phenotypes were used to obtain unadjusted and adjusted hazard ratios. The variables for adjusted model 1 were age, sex, BMI, SBP, LVEF, eGFR while variables for model 2 were: model 1 + BNP/NT-proBNP *z* scores. In the Nancy-HF cohort, Cox proportion hazard models were further adjusted for medications (ACEi/ARBs, beta-blockers and aldosterone) to assess the impact of treatment on prognosis for the identified phenotypes. The association of protein biomarkers with identified phenotypes was assessed using a multinomial logistic regression model. The *P*-values for multiple comparisons were corrected for FDR (at 5%) using the Benjamini-Hochberg correction.

A two-tailed *P*-value <0.05 was considered significant. The analyses were conducted with R [R Core Team (2020). R: A language and environment for statistical computing. R Foundation for Statistical Computing, Vienna, Austria. URL https://www.R-project.org/] and Weka software packages © (version 3.8.6).

## Results

In Nancy-HF, the mean age of study participants was 75 (±12) years and included 50% women. More than 80% of the patients had hypertension and mean LVEF was 49.0 (±15.6)%.

### Congestion phenotypes identified in the nancy-HF cohort

**Figure 1:**
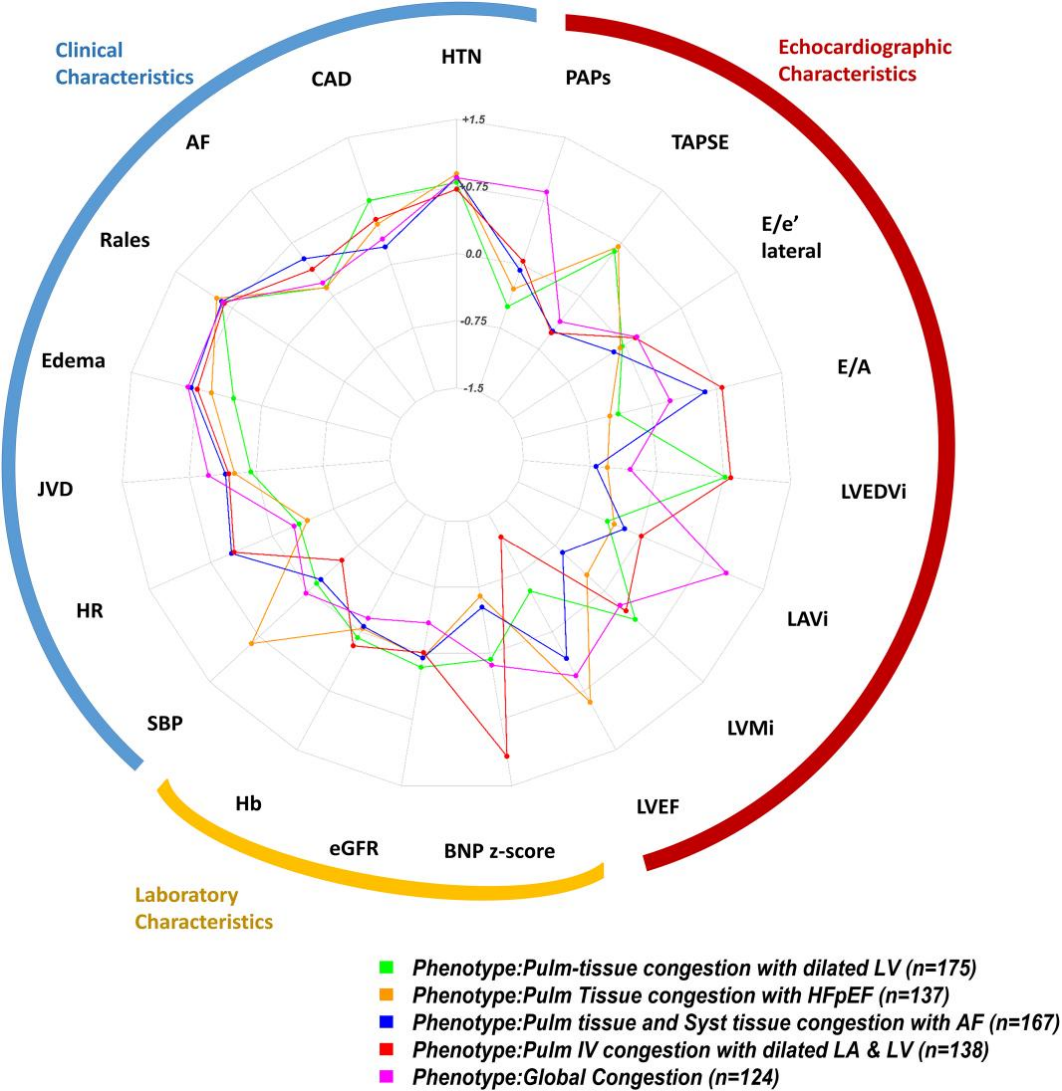
Radar plot depicting the clustering variables in nancy-HF. The axes represent the different congestion markers. The congestion phenotype is represented by polygons, with points on each axis corresponding to the standardized mean value of the respective congestion marker for that phenotype. AF, Atrial Fibrillation; BNP, Brain Natriuretic Peptide; CAD, Coronary Artery Disease; eGFR, estimated Glomerular Filtration Rate; Hb, haemoglobin; HR, Heart Rate; HTN, hypertension; JVD, Jugular Venous Distention; LAVi, Left Atrial Volume index; LVEDVi, Left Ventricular End Diastolic Volume index; LVEF, Left Ventricular Ejection Fraction; LVMi, Left Ventricular Mass index; PASP, Pulmonary artery systolic pressure; SBP, Systolic Blood Pressure; TAPSE, Tricuspid Annular Plane Systolic Excursion.

Five different congestion phenotypes of patients with AHF were identified in the Nancy-HF cohort. The top discriminative variables in the cluster analysis were BNP/NT-proBNP *z*-score, LVEF, LVEDVi, LAVi and E/A ratio (see Supplementary material online, *Figure S1S*). Baseline characteristics of the identified phenotypes are presented in *Table 1* and *Figure 1*.

**Table 1:** Baseline patient characteristics according to the identified phenotype in the nancy-HF cohort

Characteristic		Pulm-tissue congestion with dilated LV	Pulm tissue congestion with HFpEF	Pulm tissue and Syst tissue congestion with AF	Pulm IV congestion with dilated LA & LV	Global congestion	*P*-value
Pulm tissue		+ + +	+ + +	+ + +	+ +	+ + +	
Syst tissue		+	+ +	+ + +	+ +	+ + +	
Syst—IV		+	+ +	+ +	+ +	+ + +	
Pulm—IV		+	+	++	+ + +	+ + +	
	***n***	***n* = 175**	***n* = 137**	***n* = 167**	***n* = 138**	***n* = 124**	
Age	741	74 (66, 83)	79 (71, 85)	79 (70, 87)	73 (64, 81)	83 (74, 89)	<0.001
Sex = female	741	77 (44%)	71 (52%)	111 (66%)	51 (37%)	63 (51%)	<0.001
BMI	712	26 (5.4)	28.7 (6.7)	28.8 (.3)	26.6 (5.1)	25.5 (5.5)	<0.001
Hypertension	741	139 (79%)	122 (89%)	142 (85%)	99 (72%)	105 (85%)	0.002
CAD	640	102 (76%)	55 (47%)	32 (21%)	61 (53%)	36 (30%)	<0.001
AF	740	21 (12%)	16 (12%)	88 (53%)	52 (38%)	23 (19%)	<0.001
Diabetes	741	66 (38%)	70 (51%)	54 (32%)	43 (31%)	45 (36%)	0.004
Smoking	741	59 (34%)	46 (34%)	45 (27%)	47 (34%)	33 (27%)	0.4
COPD	499	12 (19%)	27 (26%)	28 (19%)	7 (9.6%)	16 (14%)	0.044
HR	708	72 (14)	70 (13)	91 (23)	90 (33)	73 (16)	<0.001
SBP	740	134 (28)	166 (33)	132 (22)	122 (28)	139 (32)	<0.001
NYHA	741						0.91
Class I		3 (1.7%)	0 (0%)	0 (0%)	2 (1.4%)	0 (0%)	
Class II		16 (9.1%)	7 (5.1%)	8 (4.8%)	12 (8.7%)	5 (4.0%)	
Class III		58 (33%)	60 (44%)	74 (44%)	64 (46%)	54 (44%)	
Class IV		98 (56%)	70 (51%)	85 (51%)	60 (43%)	65 (52%)	
Rales	499	55 (89%)	97 (95%)	131 (89%)	62 (85%)	99 (87%)	0.22
Crackles lower lung zones	499	47 (76%)	94 (92%)	109 (74%)	47 (64%)	91 (80%)	<0.001
Crackles lower- upper lung zones	499	29 (47%)	51 (50%)	52 (35%)	20 (27%)	47 (41%)	0.019
Wheezing	499	19 (31%)	31 (30%)	43 (29%)	11 (15%)	18 (16%)	0.011
Peripheral oedema	741	56 (32%)	79 (58%)	135 (81%)	102 (74%)	105 (85%)	<0.001
JVD	741	10 (5.7%)	33 (24%)	57 (34%)	42 (30%)	66 (53%)	<0.001
LVEF	702	41 (12)	63 (7)	54 (11)	30 (12)	58 (9)	<0.001
HF type	702						<0.001
HFmrEF		46 (30%)	2 (1.5%)	36 (23%)	20 (15%)	18 (15%)	
HFpEF		35 (23%)	130 (98%)	106 (67%)	8 (6.0%)	103 (84%)	
HFrEF		74 (48%)	0 (0%)	17 (11%)	105 (79%)	2 (1.6%)	
Hb	732	12.43 (2.18)	12.17 (2.07)	12.11 (2.18)	12.65 (2.37)	11.88 (2.09)	0.053
eGFR	730	59 (25)	56 (25)	57 (24)	55 (25)	47 (22)	<0.001
BNP at admission	656	1028	420	521	2002	1041	<0.001
		(610, 1556)	(271, 568)	(324, 746)	(1206, 3226)	(613, 1686)	
LVMi	671	136 (55)	104 (32)	89 (23)	130 (38)	126 (42)	<0.001
E/A	431	1.04 (0.71, 1.59)	1.03 (0.75, 1.39)	2.16 (1.62, 2.48)	2.10 (1.37, 2.92)	1.61 (1.07, 2.44)	<0.001
e′ prime velocity	320	6.30 (1.98)	7.20 (2.52)	8.57 (2.12)	6.54 (2.21)	7.25 (2.15)	<0.001
E/e′ mean	319	13.8 (5.8)	14.1 (6.5)	12.6 (4.9)	15.2 (6.2)	15.8 (5.2)	0.004
E/e′ lateral	624	11.0 (9.0, 14.9)	11.1 (8.6, 15.1)	11.1 (8.3, 13.7)	12.7 (9.7, 16.5)	12.4 (9.9, 17.2)	0.014
LAVi	669	38 (14)	41 (18)	44 (15)	50 (20)	80 (46)	<0.001
LVEDVi	663	78 (27)	41 (14)	37 (12)	79 (29)	48 (19)	<0.001
TAPSE	690	21.3 (4.4)	21.6 (4.1)	15.6 (3.8)	15.5 (4.3)	16.3 (4.2)	<0.001
PASP	630	37 (11)	40 (15)	44 (13)	45 (11)	58 (19)	<0.001
Diuretics	728	124 (72%)	103 (76%)	147 (89%)	118 (88%)	114 (94%)	<0.001
ARNi	728	8 (4.6%)	1 (0.7%)	1 (0.6%)	10 (7.4%)	2 (1.7%)	0.002
MRA	728	43 (25%)	22 (16%)	25 (15%)	37 (27%)	30 (25%)	0.031
Beta-blockers	729	147 (85%)	99 (73%)	133 (81%)	115 (85%)	91 (75%)	0.025
ACEi	729	110 (63%)	68 (50%)	73 (45%)	79 (59%)	56 (46%)	0.003
ARA	728	28 (16%)	43 (32%)	40 (24%)	14 (10%)	22 (18%)	<0.001

ACEi, Angiotensin Converting Enzyme inhibitor; AF, Atrial Fibrillation; ARA, Angiotensin Receptor Antagonist; ARNi, Angiotensin and Neprilysin Inhibitor; BMI, Body Mass Index; BNP, Brain Natriuretic Peptide; CAD, Coronary Artery Disease; COPD, Chronic Obstructive Pulmonary Disease; eGFR, estimated Glomerular Filtration Rate; Hb, haemoglobin; HF, heart failure; HFmrEF, HF with mildly-reduced Ejection Fraction; HFpEF, HF with preserved Ejection Fraction; HFrEF, HF with reduced Ejection Fraction; HR, Heart Rate; JVD, Jugular Venous Distention; LA, Left Atrium; LAVi, Left Atrial Volume index; LV, Left Ventricle; LVEDVi, Left Ventricular End Diastolic Volume index; LVEF, Left Ventricular Ejection Fraction; LVMi, Left Ventricular Mass index; MRA, Mineralocorticoid Receptor Antagonist; NYHA, New York Heart Association; PASP, Pulmonary artery systolic pressure; Pulm, Pulmonary; SBP, Systolic Blood Pressure; TAPSE, Tricuspid Annular Plane Systolic Excursion.

Categorical variables presented as *n* (%), continuous variables as mean (SD) if distribution normal or Median (Q1, Q3) if distribution non-normal.

The key characteristics of the five phenotypes are as follows:

- ‘Pulm-tissue congestion with dilated LV’ (PTC-dilated LV; *n* = 175): patients with a high likelihood of CAD, frequent rales, all ranges of LVEF (range 16–68%), high LVMi and high LVEDVi.
- ‘Pulm tissue congestion with HFpEF’ (PTC-HFpEF; *n* = 137): patients with high SBP, frequent rales, predominantly HFpEF (LVEF range 45–77%) with small to normal LVEDVi.
- ‘Pulm tissue and Syst tissue congestion with AF’ (PTC, STC-AF; *n* = 167): high likelihood of AF, frequent rales and peripheral oedema, all ranges of LVEF (range 21–82%), high E/A, lower TAPSE and small LVEDVi.
- ‘Pulm IV congestion with dilated LA and LV’ (PIVC-dilated LA and LV; *n* = 138): mostly men, predominantly HFrEF (LVEF range 11–70), highest BNP, low TAPSE, dilated LA and LV.
- ‘Global’ congestion (*n* = 124): frequently raised JVD, peripheral oedema, predominantly HFpEF and HFmrEF (LVEF range 38% to 81%), severely dilated LA, lower TAPSE and high PASP.

### Models predicting cluster membership

The RF model exhibited 77.3% sensitivity, 93.7% specificity, and 79.4% precision (see Supplementary material online, *Figure S2S*). The decision tree and support vector machine models had a lower predictive capacity than the RF model (∼68% sensitivity, 92% specificity, and 68% precision see Supplementary material online, *Figures S3S*–*S5S*). XGBoost had similar prediction parameters as RF model however; cluster characteristics of phenotypes in the validation cohort were different from derivation cohort (data not shown). Cluster memberships were hence predicted using RF in the subsequent steps of the analysis in the BIOSTAT-CHF (validation) cohort.

### Predicted congestion phenotypes in BIOSTAT- CHF

In BIOSTAT-CHF, nearly 30% of participants were women and mean age was 70 (±12) years. The RF model (constructed in Nancy-HF) identified similar congestion profiles in BIOSTAT-CHF (see Supplementary material online, *Table S1S*).

In the validation cohort, the majority of patients were classified as ‘PTC-dilated LV’ (*n* = 2796/4254, 65.7%), followed by ‘PIVC-dilated LA & LV’ (*n* = 956/4254, 22.5%) and ‘PTC, STC-AF’ (*n* = 384/4254). Fewer patients were identified in the ‘PTC-HFpEF’ (*n* = 83) and ‘Global’ congestion phenotypes (*n* = 35). The distribution pattern of clinical characteristics, congestion markers and echocardiography variables in the validation cohort was similar to the derivation cohort.

### Pathways linked to protein biomarkers associated with congestion phenotypes

Compared with ‘PTC-dilated LV’, 287 proteins were significantly associated with other clusters (see Supplementary material online, *Figure S6S* and *Table S2S*). Of the 287 proteins, 25 proteins were associated with all of the clusters, while some biomarkers were identified in only one cluster: 3 proteins with ‘PTC-HFpEF’ (all down-regulated), 7 proteins significantly associated with ‘PTC, STC-AF’ (2 up-regulated: 5 down-regulated), 49 proteins significantly associated with ‘PIVC-dilated LA & LV’ (32 up-regulated: 17 down-regulated) and 1 significantly associated with the ‘Global’ congestion (up-regulated) phenotype (see Supplementary material online, *Table S2S*).

**Figure 2:**
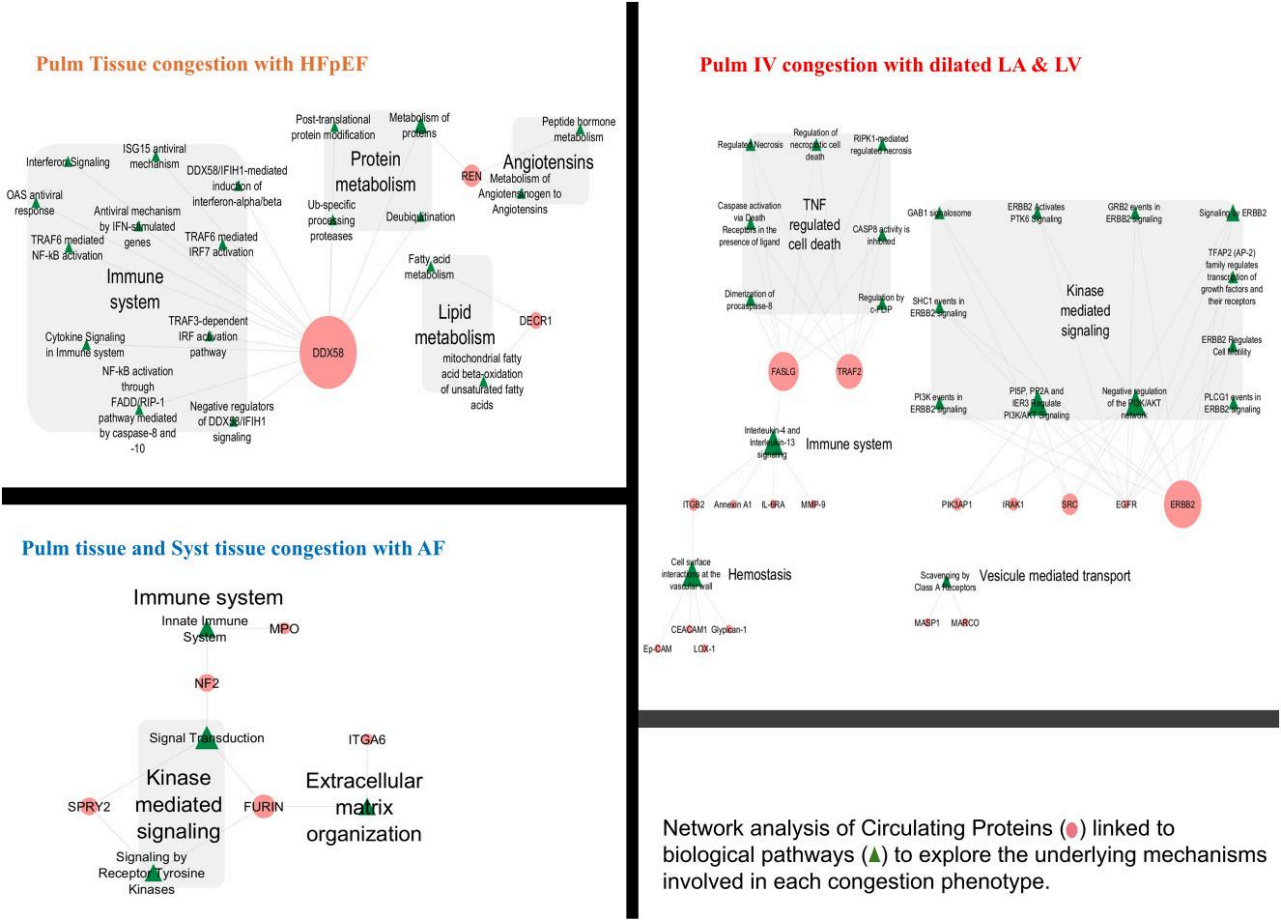
Biological pathways linked to protein biomarkers significantly associated with each phenotype in BIOSTAT-CHF. The size of the protein node (circles) corresponds to the number of connected pathways with the respective protein while the size of the pathway node (triangles) corresponds to the number of proteins linked to the pathway. All pathways linked to the identified proteins are presented for ‘Pulm Tissue congestion with HFpEF’. Pathways connecting at least 2 proteins are presented for ‘Pulm tissue and Syst tissue congestion with AF’. Over-represented pathways are presented for ‘Pulm IV congestion with dilated LA & LV’.

All protein-biological pathways characterized via the network analysis are presented in *Figure 2*. ‘PTC-HFpEF’ was associated with pathways regulating the immune system and metabolism of angiotensin, proteins and lipids. DDX58 emerged as a central node regulating these latter pathways in this phenotype. ‘PTC, STC-AF’ was associated with the innate immune system, extracellular matrix organisation and kinase-mediated signalling. The ‘PIVC-dilated LA & LV’ phenotype was associated with kinase-mediated signalling, TNF-regulated cell death, immune system, hemostasis and vesicle-mediated transport. ERBB2, FASLG, TRAF2 emerged as the key nodes in this phenotype. The ‘Global’ congestion phenotype was associated with only Gal-4 protein and no associated protein pathways were identified in the Reactome database.

### Association of congestion phenotypes with survival in the nancy-HF and BIOSTAT-CHF cohorts

In Nancy-HF, 361 events (155 HHF and 272 deaths) were observed over a median follow-up of 17.1 [6.3, 29.0] months. Stratified analyses revealed that, as expected, the distribution of phenogroups varied across age, sex, and LVEF category strata. Importantly, the risk stratification properties of the phenogroups were largely preserved within these strata (see Supplementary material online, *Figure S7S*).

**Figure 3:**
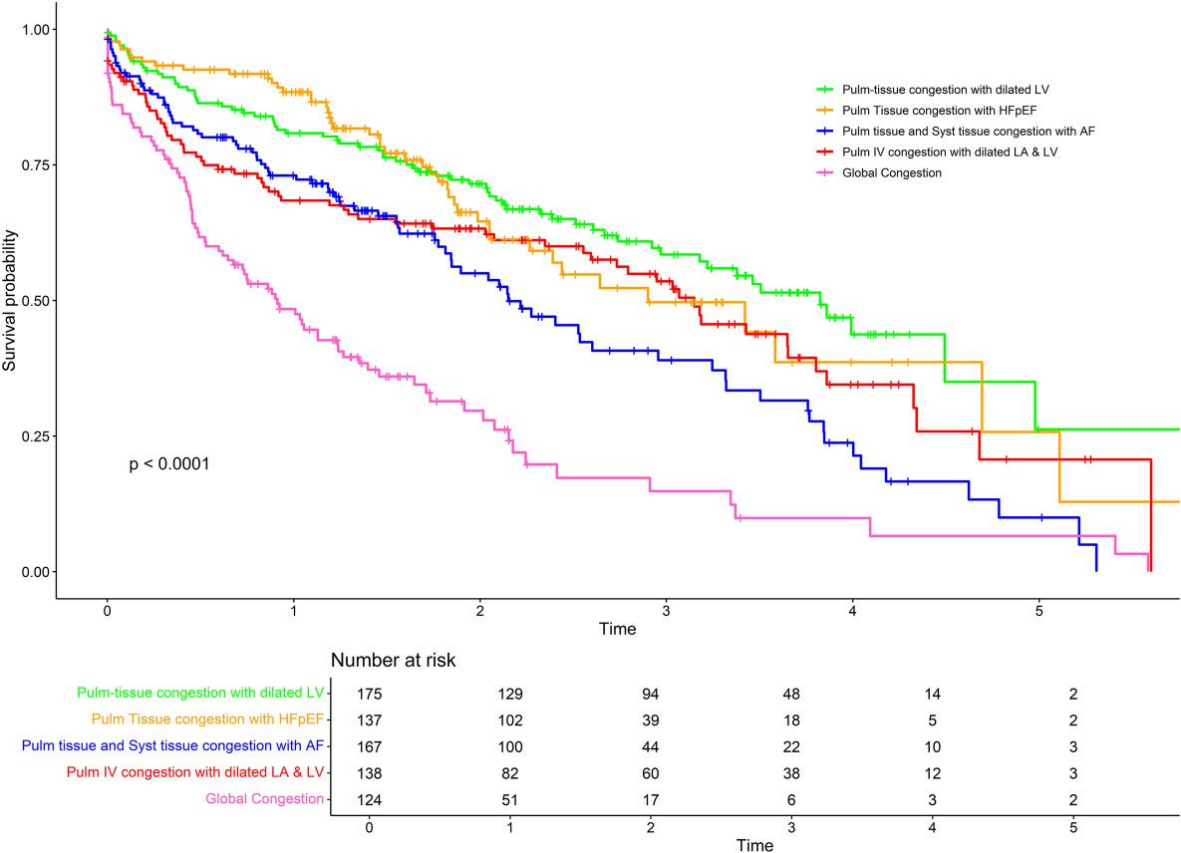
Kaplan-Meier curves according to identified phenotype in nancy-HF.

‘PTC-dilated LV’ and ‘PTC-HFpEF’ had a higher survival probability compared with the other phenotypes (*Figure 3*), followed by ‘PTC, STC-AF’ and ‘PIVC-dilated LA & LV’ whereas the ‘Global’ congestion phenotype had the worst prognosis. The adjusted association (M1) with primary outcome was HR = 1.50 (1.01–2.23), *P* = 0.044 for ‘PTC, STC-AF’, HR 1.37 (0.94–2.00), *P* = 0.099 for ‘PIVC-dilated LA & LV’ (*Table 2*) and HR = 2.38 (1.61–3.52), *P* < 0.0001 for ‘Global’ congestion compared with ‘PTC-dilated LV’. Similar results were observed after adjusting for BNP *z*-score (*Table 2*) and medications (see Supplementary material online, *Table S3S*).

**Table 2:** Association of identified phenotype with composite outcome in the nancy-HF and BIOSTAT-CHF cohorts

Phenotype	*n* events/*n*	Nancy-HF		*n* events/*n*	BIOSTAT-CHF	
		HR (95% CI)	*P*-value		HR (95% CI)	*P*-value
Unadjusted	361/741			1702/4109		
Pulm-tissue congestion with dilated LV		Ref.			Ref.	
Pulm Tissue congestion with HFpEF		1.07 (0.74–1.55)	0.73		0.91 (0.62–1.34)	0.64
Pulm tissue and Syst tissue congestion with AF		1.80 (1.31–2.48)	0.0003		1.49 (1.27–1.74)	<0.0001
Pulm IV congestion with dilated LA & LV		1.40 (1.01–1.96)	0.045		1.76 (1.58–1.96)	<0.0001
Global congestion		3.52 (2.56–4.85)	<0.0001		2.96 (1.98–4.45)	<0.0001
Model 1*	330/668			1519/3705		
Pulm-tissue congestion with dilated LV		Ref.			Ref.	
Pulm Tissue congestion with HFpEF		0.98 (0.60–1.59)	0.93		0.78 (0.49–1.24)	0.29
Pulm tissue and Syst tissue congestion with AF		1.50 (1.01–2.23)	0.044		1.13 (0.92–1.41)	0.25
Pulm IV congestion with dilated LA & LV		1.37 (0.94–2.00)	0.099		1.65 (1.46–1.87)	<0.0001
Global congestion		2.38 (1.61–3.52)	<0.0001		1.82 (1.17–2.83)	0.008
Model 2*	303/605			1082/2455		
Pulm-tissue congestion with dilated LV		Ref.			Ref.	
Pulm Tissue congestion with HFpEF		1.16 (0.68–1.98)	0.58		0.74 (0.45–1.20)	0.22
Pulm tissue and Syst tissue congestion with AF		1.74 (1.13–2.67)	0.012		1.05 (0.83–1.32)	0.69
Pulm IV congestion with dilated LA and LV		1.23 (0.81–1.86)	0.33		1.43 (1.21–1.70)	<0.0001
Global congestion		2.41 (1.60–3.63)	<0.0001		1.64 (1.04–2.58)	0.033

Reference: Pulm-tissue congestion with dilated LV.

Model 1: adjusted for age, sex, BMI, SBP, LVEF, eGFR.

Model 2: Model M1 + NT-proBNP *z* score.

The outcome was a composite of re-hospitalisation for HF or death in the derivation cohort (Nancy-HF) and a composite of death and hospitalisation in validation cohort (BIOSTAT-CHF).

**Figure 4:**
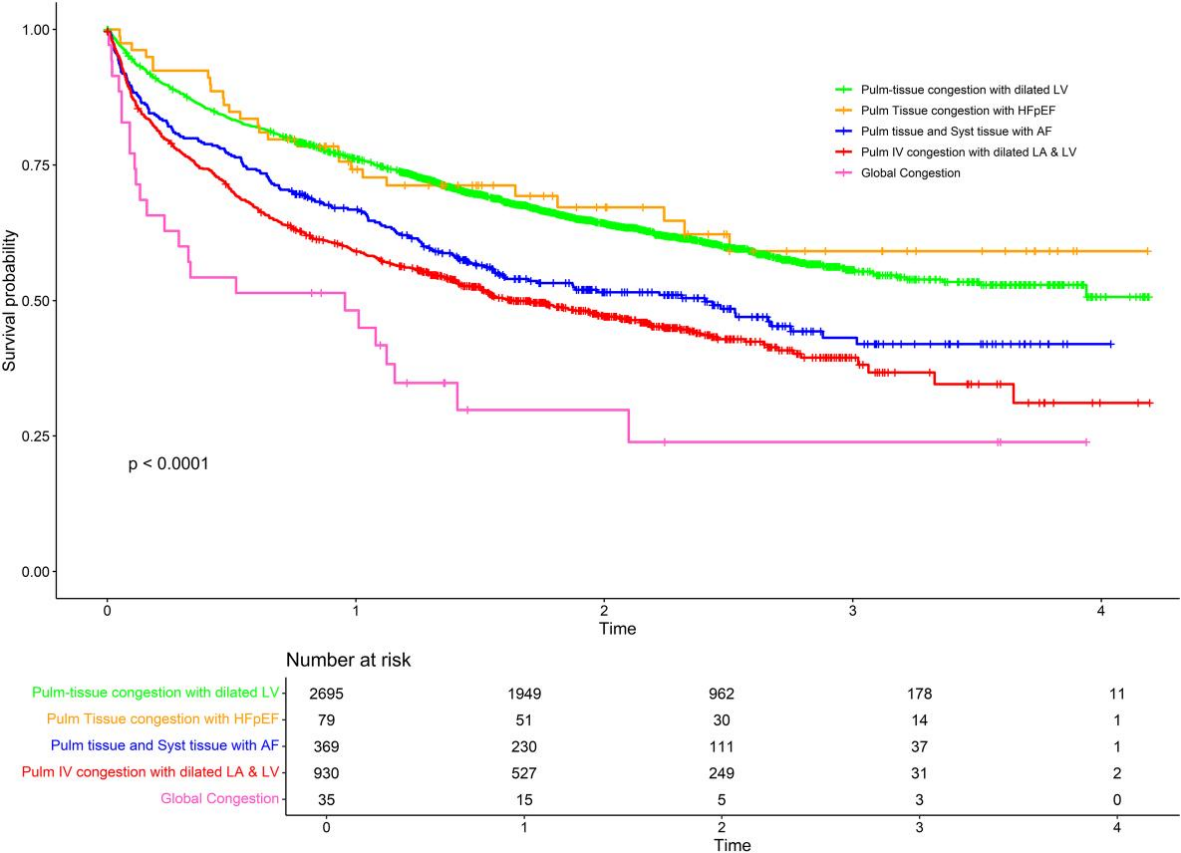
Kaplan-Meier curves according to predicted phenotype in BIOSTAT-CHF. The outcome was a composite of hospitalisation for HF or death in the derivation cohort and the validation cohort (BIOSTAT-CHF).

In the BIOSTAT-CHF cohort, the ‘PTC-dilated LV’ and ‘PTC-HFpEF’ phenotypes had a better survival prognosis, followed by the ‘PTC, STC-AF’, ‘PIVC-dilated LA & LV’ and ‘Global’ congestion phenotypes (*Figure 4*) over a median follow-up time of 18.6 [8.5, 27.3] months. The risk of primary outcome was significantly higher in ‘PIVC-dilated LA & LV’ and ‘Global’ congestion in Model M1 [HR =1.65 (1.46–1.87)], *P* < 0.0001 and [HR = 1.82 (1.17–2.83)], *P* = 0.008, respectively but not in the ‘PTC, STC-AF’ phenotype. (*Table 2*).

## Discussion

The unsupervised machine learning analysis applied in the present study identified five distinct phenotypes based on clinical characteristics and type and severity of congestion markers in AHF patients. These phenotypes could be predicted effectively using an RF model (Graphical abstract). More importantly, each congestion profile had a relatively distinct protein profile and involved different biological pathways, hence shedding new light on our understanding of congestion in acute settings. Our findings suggest that classifying AHF using a machine learning model based on clinical and congestion parameters could prove useful in categorising the patients and predicting risk in future studies aimed at exploring individualized therapies in light of the different biological backgrounds related to each congestion phenotype.

### Congestion phenotypes in AHF

Routinely available clinical variables (hypertension, CAD, AF, rales, peripheral oedema, JVD, heart rate, SBP), laboratory variables (haemoglobin, eGFR, BNP) as well as echocardiography variables (LVEF, LVMi, LAVi, LVEDVi, TAPSE, PASP, E/e′ lateral, and E/A ratio) were selected to conduct the clustering analysis herein. Demographic characteristics such as age, sex or BMI were not included in order to maintain focus on identifying the congestion profiles as these variables significantly influence cluster designation. Factors such as CAD, AF, LVMi, and TAPSE were considered as they provide insights into the etiopathogenesis of HF, prognosis and aid in the adequate assessment of congestion. Nevertheless, the phenotypes obtained after clustering analysis were, to some extent, congruous with certain phenotypes reported in previous studies, including demographic profiles and other comorbidities.^25,26^ The phenogroups ‘PTC-dilated LV’, PTC, ‘STC -AF’, and ‘Global’ congestion were comparable to profiles identified as ‘Young’, ‘Elderly/AF’, and ‘Diabetes and CKD’ (despite the differences in age and frequency of comorbidity) in a recent study which identified AHF phenotypes based on comorbidities.^25^ Moreover, these phenogroups had the best, intermediate and worst prognosis respectively in both studies. Our findings suggest that different AHF phenotypes may overlap with congestion patterns, possibly due to the impact of comorbidities on the development of congestion (e.g. sodium avidity).

The comorbidity and echocardiographic profiles of the ‘PTC-dilated LV’, ‘PTC, STC-AF’, and ‘PIVC-dilated LA & LV’ phenotypes are partially concordant with the clusters identified in previous studies assessing a broad spectrum of HF patients.^16,27,28^ ‘PTC-HFpEF’ and ‘Global’ congestion clusters had predominant HFpEF subtypes and similar proportions of NYHA class III/IV at baseline. However, patients in the ‘Global’ congestion phenotype were older (mean age 83 vs. 79) and had higher left heart volumes, higher filling pressure and lower TAPSE compared with the ‘PTC-HFpEF’ phenotype. A similar distinction was also observed in patients with HFpEF when unsupervised machine learning analysis was conducted using demographic and echocardiography markers^29^ emphasising the role of congestion markers in distinguishing these phenotypes. Moreover, the ‘Global’ congestion phenotype incurred higher risk even after adjusting for natriuretic peptide levels in both the Nancy-HF and BIOSTAT-CHF cohorts, thus confirming that within HFpEF phenogroups with similar metabolic comorbidities, congestion phenotypes could also influence prognosis. It is possible that these two groups require distinct management strategies to improve congestion and overall prognosis. Our findings align with a previous study which identified HF clusters using clinical congestion variables such as JVD, leg oedema, S3, crackles, and orthopnea in AHF.^30^ In effect, using clustering analysis on physical signs of congestion, Niimi *et al*.^30^ found that the cluster primarily characterized by congestion with JVD was associated with a higher risk of adverse outcome compared with clusters with no signs of congestion.^30^ However, ‘PTC, STC -AF’ was associated herein with increased risk in the Nancy-HF cohort but not in the BIOSTAT-CHF cohort, probably because the validation cohort had relatively preserved left atrial volume and E/A ratio despite their similarity in terms of age and other characteristics such as eGFR, SBP and LVEF.

### The biological background of congestion phenotypes

The differences observed in the five congestion phenotypes were reflected in the network analysis of proteins associated with each individual phenotype. ‘PTC-HFpEF’ was significantly associated with DDX58 (retinoic acid-inducible gene I), REN (Renin) and DECR1 (2,4-dienoyl-CoA reductase) proteins, which were down-regulated compared with the ‘PTC-dilated LV’ phenotype. REN is an established biomarker having prognostic significance in heart failure, whereas DDX58 and DECR1 are relatively new proteins identified in the present study in association with congestion and HF. DDX58 is central in regulating immune system pathways^31^ while DECR1 plays a key role in fatty acid metabolism in myocardial cells.^32^ A previous mechanistic study in mice demonstrated that excessive fatty acids reduced DDX58 protein expression, thereby attenuating the autophagic responses required for clearing toxic lipid inclusion bodies, which may lead to inflammation-mediated cell death.^33^

In the ‘PTC, STC-AF’ phenotype, FURIN protein was linked with pathways related to the immune system, kinase-mediated signalling and extracellular matrix organisation. FURIN, a pro convertase protein, converts NTproBNP to active forms, cleaves pro-protein PCSK 9 and plays a role in blood pressure regulation and atherosclerotic plaque formation.^34^ Moreover, an experimental study demonstrated that FURIN overexpression was associated with atrial remodelling in early HF.^35^ FURIN could hence be a potential biomarker for identifying patients with early AF and HF given its role in atrial remodelling and overall early development in atrio-ventricular junctions.^36^ Dysregulated ITGA6 and MPO, which are also involved in pathways linked to the ‘PTC, STC-AF’ phenotype, are associated with increased cardiac fibrosis and pressure overload as well as systolic and diastolic dysfunction in HF.^37–39^

‘PIVC-dilated LA and LV’ was associated with kinase-mediated signalling, TNF-regulated cell death, hemostasis, immune system and vesicle-mediated transport. Network analysis showed that ERBB2 signalling, FASLG (member of the TNF superfamily) and TRAF2 (member of the TNF receptor associated factor) were focal points of the network. Our findings partially align with previous studies, which identified HFrEF as more prominently associated with pathways of kinase-mediated signalling, TNF-mediated cell response^40,41^ and hemostatsis.^42^ Dysregulation of EGFR:erbB2 axis (epidermal growth factor receptor and EGFR tyrosine kinase) is associated with HF severity and regulates apoptosis and myocyte hypertrophy.^43,44^ It plays an important role in the myocardial response to stress overload and may represent a potential therapeutic target.^43,45^ Remarkably, the ‘Global’ congestion phenotype was solely associated with Gal-4 protein only, indicating a large overlap of dysregulated proteins with other phenotypes. Gal-4 was not associated with any particular biological pathway in the Reactome. Gal-4, a transporter of apical proteins such as protease dipeptidyl peptidase-4,^46^ has been shown to be linked with an increased risk of incident HF^47^ as well as increased risk of adverse events in severe HF^48^ and HF with obesity and diabetes in recent studies.^46^ The other members of the galectin family are associated with the inflammatory immune response in cardiovascular metabolic diseases; however, studies on Gal-4 are limited, and further research is warranted to elucidate its role in fibrosis and congestion in HF.

While immune system dysregulation was a recurring theme across all phenotypes, both the associated proteins and underlying pathways, on the other hand, varied according to phenotype. Although inflammation and immune responses have been associated with cardiovascular diseases for the past three decades, targeting specific immune molecules has failed to show favourable outcomes to date.^49^ The present findings further emphasize the heterogeneity in immune response depending on HF subtype, comorbidities and type of congestion, and that each phenotype may require a more personalized treatment approach. The network analysis performed herein enabled providing meaningful insight into the key mechanisms underpinning congestion phenotypes of HF. A greater understanding of these pathways could prove highly useful in potentially identifying diagnostic and therapeutic targets.

### Assigning clusters to identified phenotypes using the RF model and validation

While cluster assignment appears clinically intuitive based on differences in clinical characteristics, particularly given the overlap of clinical features, an AI-based model may better assign phenotypes by analyzing the grouping of several clinical and echocardiographic characteristics that may not be possible clinically. Our RF model exhibited 77% accuracy in predicting the cluster sub-group. It should be noted that the accuracy of the RF model in assigning clusters was much higher for the ‘PTC-dilated LV’ and ‘PTC, STC -AF’ phenotypes while lower for the ‘Global’ congestion phenotype. The usefulness of this RF model was moreover validated in the BIOSTAT-CHF cohort, thus confirming the prognostic value of using the RF model for patient cluster prediction which can be accessed through the (Ai-Cong-HF) webpage available to all clinicians during their consultations, (https://cic-p-nancy.fr/ai-cong-hf/). As this model requires few clinical and echocardiographic variables, the model can easily be used in clinics or in future studies to study the different AHF phenotypes.

In addition to RF model, we explored XGBoost as AI-model to predict clusters. XGBoost had similar prediction performance as RF model (F1 measure ∼ 78%). However, in this analysis, the characteristics of the predicted phenotypes in the validation dataset were not congruent with the characteristics of the derivation cohort when XGBoost model was used. Therefore, the RF models presented in this study offer superior validity. This is in line with published data suggesting that RF models are generally more robust against overfitting compared with XGBoost models.^50^

### Clinical implications of our findings

These findings confirm that patients may be sub-classified into similar congestion profiles within a very heterogeneous AHF population. While most previous studies conducted unsupervised clustering to identify AHF phenotypes, the present model used only variables contributing to congestion assessment to identify congestion phenotypes, thereby contributing toward a better understanding of clinical and cardiac structural and functional differences associated with different congestion phenotypes in patients with AHF. Among the identified proteins and pathways, several well-documented pathways and proteins were found including REN, MPO and members of TNF and associated families, but also relatively new, less established proteins in the HF domain such as DDX58, FURIN, ERBB2, and Gal-4. These protein pathways provide clues for designing more innovative clinical studies assessing novel HF therapies. Furthermore, clinicians may use the weblink to assess their patients’ congestion profile and prognosis. This tool offers a preliminary framework that permits labelling patients in a routine clinical setting and which may, in the future, contribute towards overall clinical management of these patients, as previously done.^51^ Whether decongestive strategies should be adapted to the congestion profiles predicted by our model warrants investigation in subsequent trials.

#### Limitations

This is a retrospective study; hence, the findings must be considered as hypothesis-generating. Missing data could have impacted the cluster designation and RF model. The treating physician assessed the clinical congestion signs and echocardiographic parameters, which may be biased due to inter-observer variability. Data on inferior vena cava diameter were not available, and thus intravascular congestion status based on JVD could not be reliably assessed. In addition, the Nancy-HF cohort consists of single-centre patient data, with a homogenous patient population with regard to demographic characteristics and access to healthcare. However, validation in a large multicenter European cohort (BIOSTAT-CHF) addresses this limitation to some degree. While the results were validated in another European cohort, these findings may not be valid for other populations. Although E/e′, TAPSE, and PAPS were not in the top five discriminants of the RF model, their unavailability in the BIOSTAT-CHF cohort could have impacted the cluster classification to some extent. In addition,the two populations had different demographic profiles (age of patients, proportion of women), which could be a reason for the observed differences in the risk associated with ‘PTC, STC-AF’ and ‘PIVC-dilated LA and LV’ phenogroups. We could not compare the classification done using clustering analysis with that of Forrester classification because data on perfusion status was not collected in the Nancy-HF cohort. Furthermore, some of the prognostic markers such as hypertrophic cardiomyopathy were not considered in the clustering analysis because of limited data availability. Furthermore, some of the prognostic markers such as hypertrophic cardiomyopathy were not considered in the clustering analysis because of limited data availability. However, given the relative rarity of hypertrophic cardiomyopathy and the inclusion of LVMi in the clustering process, we believe this limitation is unlikely to have a significant impact on the results. Also, Cox proportional hazard models could not be adjusted for ARNi and SGLT2i as the data was collected between 2015 and 2019 when ARNi were prescribed in 3% of the population and SGLT2i were available in France after April 2020.

## Conclusion

In patients with AHF, clustering techniques identified five clinical congestion profiles associated with both long-term clinical risk and differences in biomarkers, suggesting potential different underlying pathophysiologies. Phenotyping patients according to their congestion profile could prove useful in further studies assessing novel therapeutic approaches in AHF.

## Supplementary Material

ztaf065_Supplementary_Data

## Data Availability

Data that supports the findings of this study are available on the reasonable request to authors and with permission of the sponsors.
