# Supplementary material for: Identifying congestion phenotypes using unsupervised machine learning in acute heart failure
Source: Eur Heart J Digit Health. 2025 Jul 15;6(5):907–18. doi: 10.1093/ehjdh/ztaf065 (PMC12450512; doi:10.1093/ehjdh/ztaf065)
Supplement: ztaf065_Supplementary_Data [file ztaf065_supplementary_data.docx]

**Supplementary data**

**Figure 1S: Plot showing discriminative power of variables in the LCM model**

*
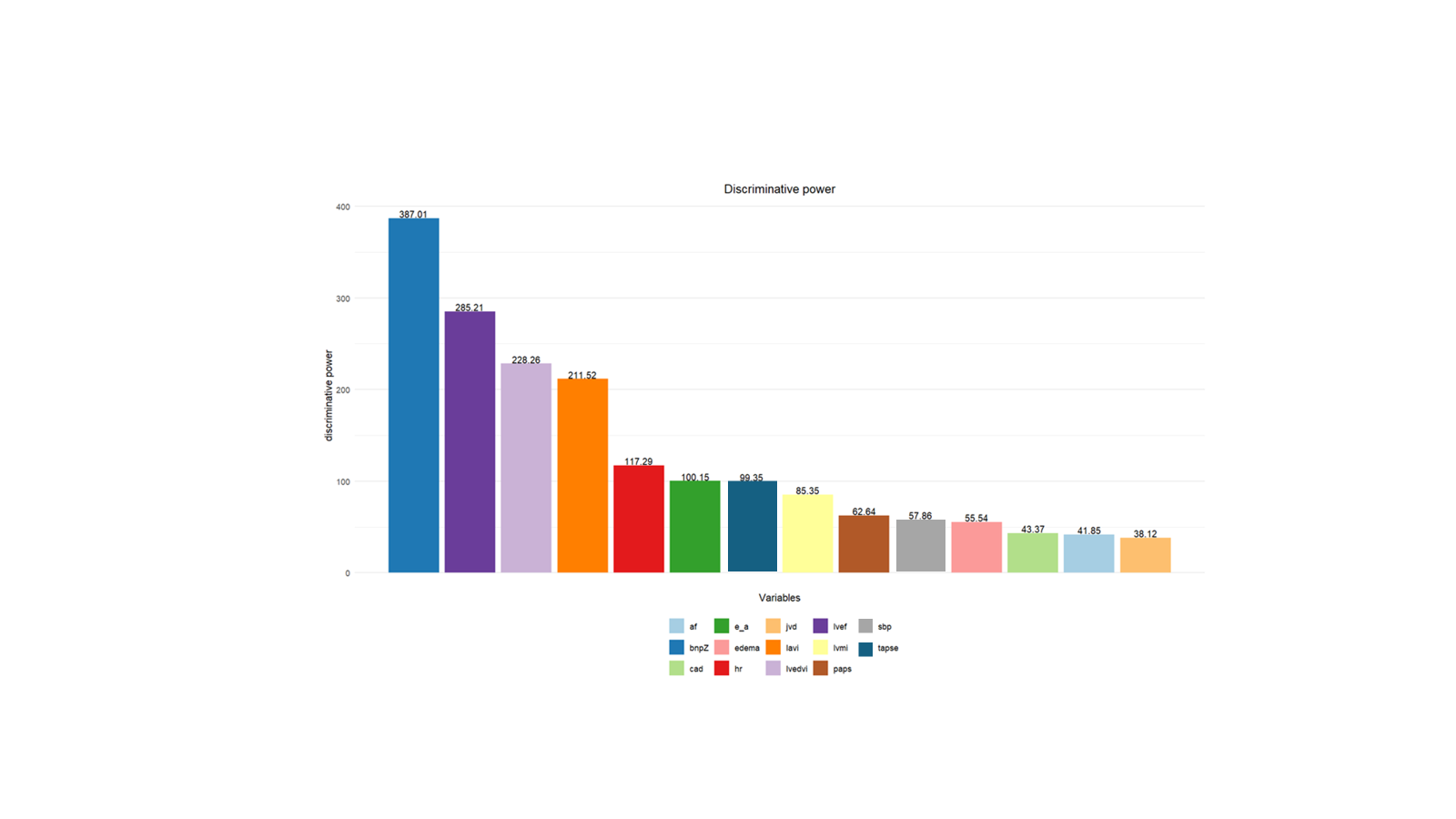
*

**R plot can fill a maximum of 12 colors. Hence, colors for SBP and TAPSE were added manually.*

**Figure 2S: performance characteristics of the random forest model**

Random forest: iterations 250


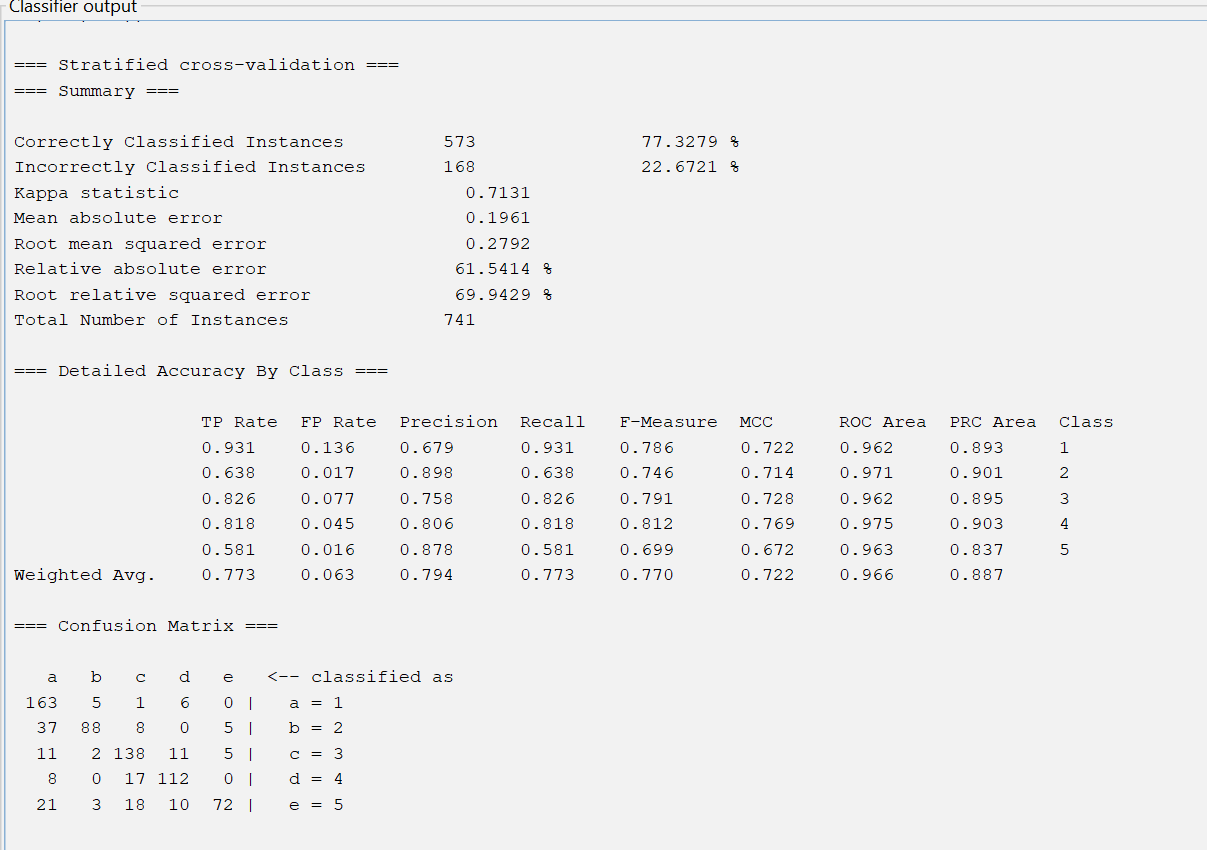


**
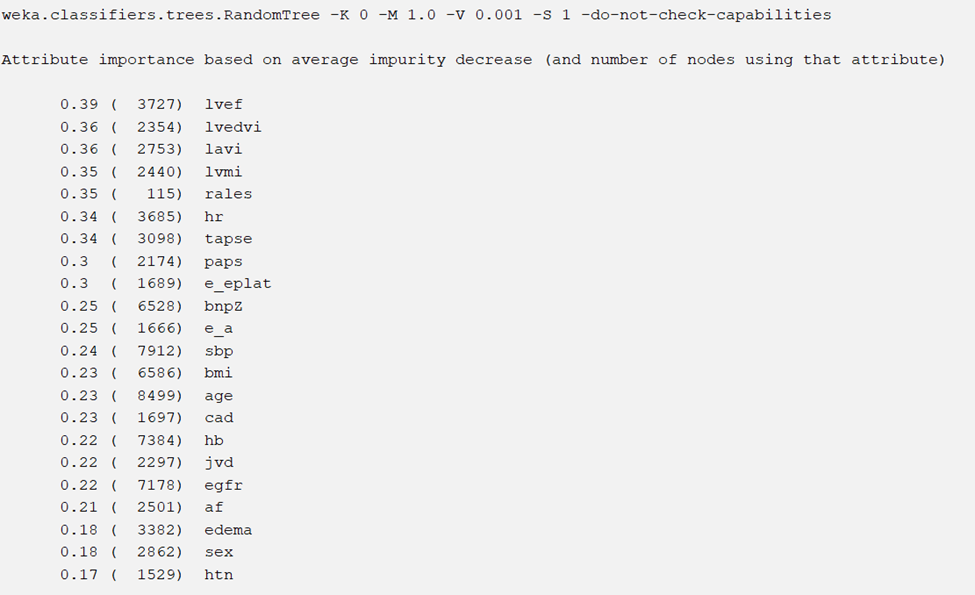
**

Decision Tree:

**Figure 3S: Detailed decision tree with minimal instances per leaf =7**


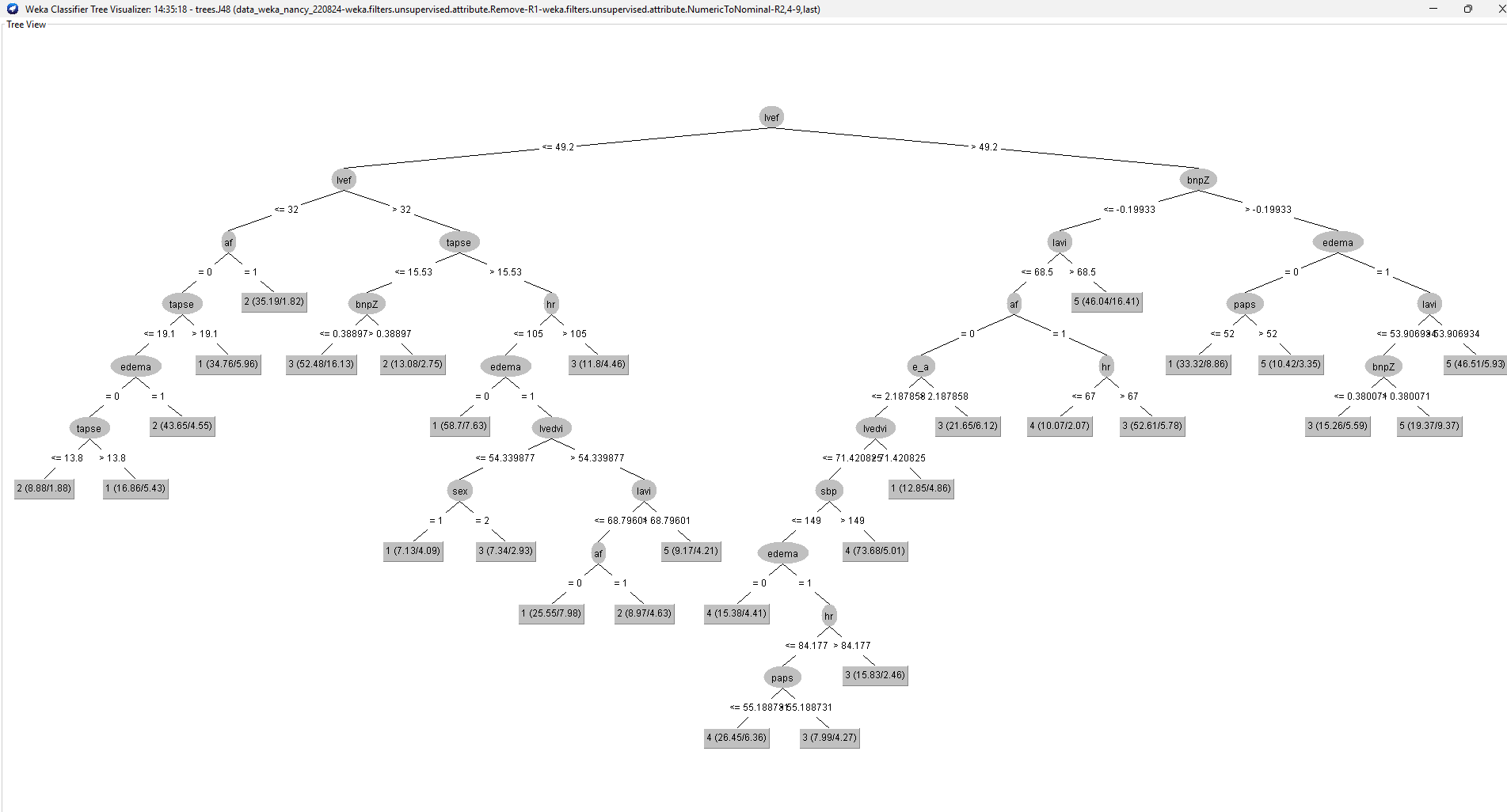


The first number in brackets corresponds to the total number of classified objects and the 2^nd^ number corresponds to misclassified objects.

**Figure 4S: Decision tree: simplified with logBNP**


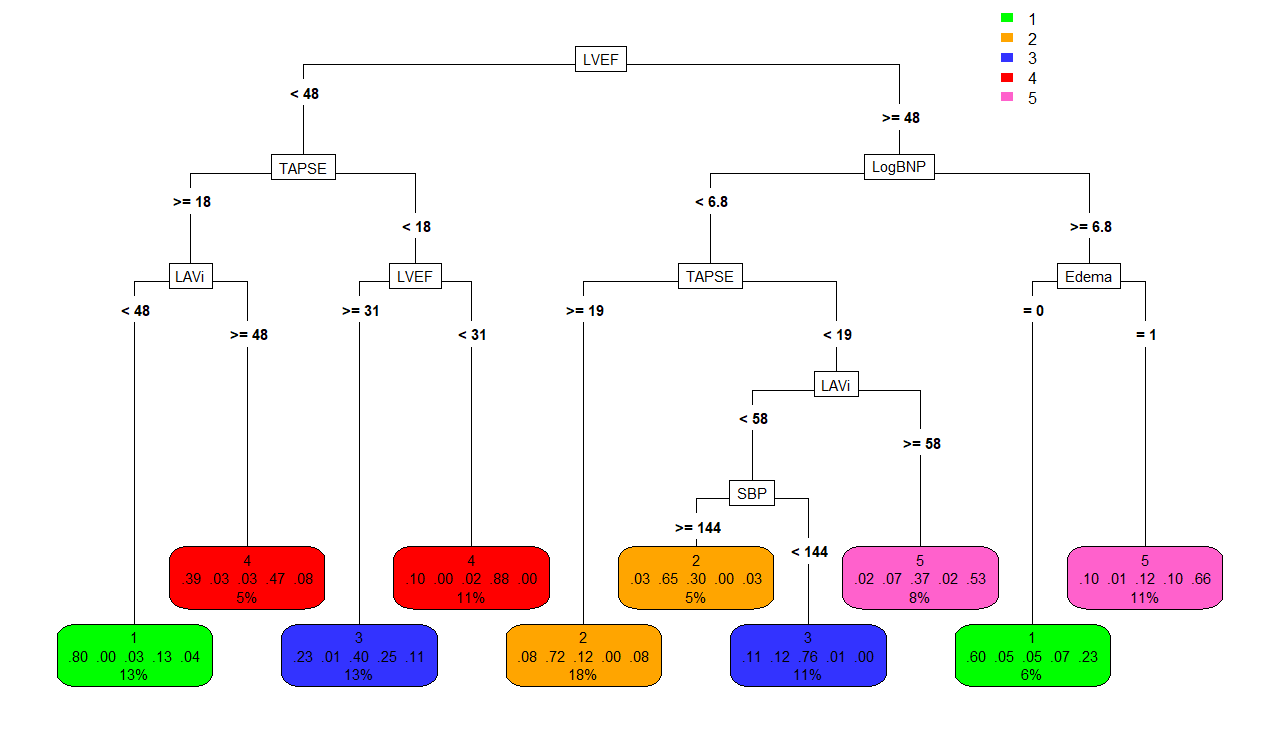


Simplified decision tree with log BNP instead of BNP z-score.

The number corresponding to the cluster number represents the correctly classified patients in the leaf. The reported percentages represent the percentage of patients in each leaf.

For example, in cluster 1 (green box): 80 % patients were of cluster 1, 0% patients were of cluster 2, 3% patients were of cluster 3, 13% patients were of cluster 4 and 4% patients were of cluster 5. A total of 13% patients from the dataset were classified as cluster 1 (sum may not be 100% due to rounding).

**Figure 5S: performance characteristics of the support vector machine model**


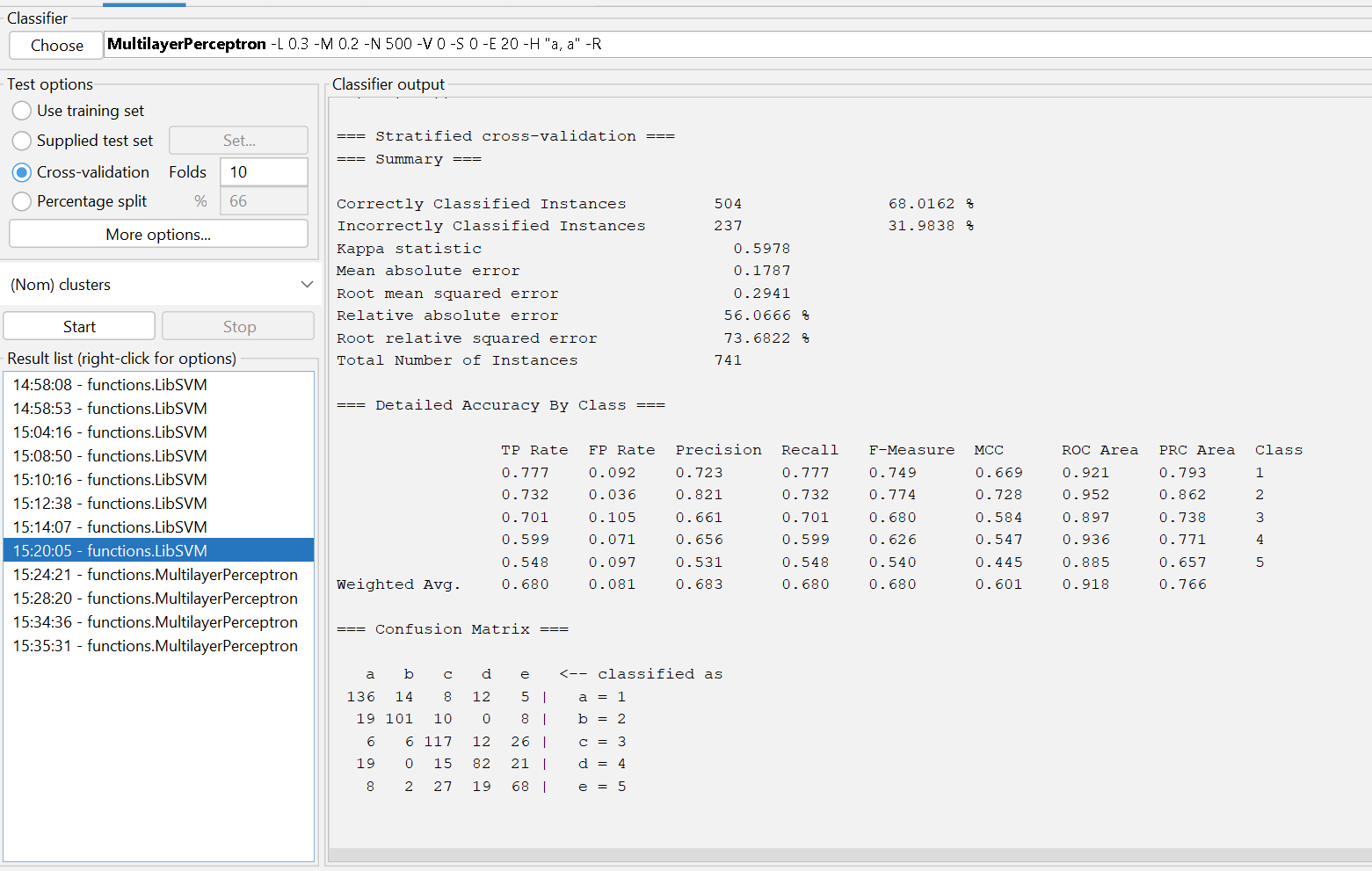


**Table 1S: Baseline characteristics of phenotypes identified in BIOSTAT-CHF using the RF model**

| Characteristic |  | Pulm-tissue congestion with dilated LV | Pulm Tissue congestion with HFpEF | Pulm tissue and Syst tissue congestion with AF | Pulm IV congestion with dilated LA & LV | Global congestion | p-value ^1^ |
| --- | --- | --- | --- | --- | --- | --- | --- |
|  | N | N = 2,796 | N = 83 | N = 384 | N = 956 | N = 35 |  |
| Age | 4,254 | 71 (63, 78) | 76 (69, 83) | 78 (70, 83) | 70 (62, 77) | 84 (81, 88) | <0.001 |
| Sex | 4,254 | 738 (26%) | 45 (54%) | 250 (65%) | 210 (22%) | 20 (57%) | <0.001 |
| BMI | 4,181 | 28.2 (5.6) | 30.3 (6.3) | 30.7 (7.5) | 27.8 (6.0) | 28.2 (5.0) | <0.001 |
| Hypertension | 4,247 | 1,672 (60%) | 73 (88%) | 268 (70%) | 533 (56%) | 30 (86%) | <0.001 |
| CAD | 3,669 | 1,748 (73%) | 57 (85%) | 170 (59%) | 496 (56%) | 15 (65%) | <0.001 |
| AF | 4,240 | 990 (36%) | 18 (22%) | 267 (70%) | 603 (63%) | 25 (71%) | <0.001 |
| Diabetes | 4,245 | 878 (31%) | 36 (43%) | 128 (33%) | 327 (34%) | 11 (31%) | 0.12 |
| Smoking | 4,239 |  |  |  |  |  |  |
| None |  | 1,174 (42%) | 38 (46%) | 223 (59%) | 375 (39%) | 14 (42%) |  |
| Past |  | 1,220 (44%) | 36 (43%) | 124 (33%) | 426 (45%) | 19 (58%) |  |
| Current |  | 396 (14%) | 9 (11%) | 34 (8.9%) | 151 (16%) | 0 (0%) |  |
| COPD | 4,238 | 458 (16%) | 13 (16%) | 82 (22%) | 194 (20%) | 8 (23%) | 0.016 |
| NYHA | 4,183 |  |  |  |  |  |  |
| I |  | 58 (2.1%) | 1 (1.2%) | 4 (1.1%) | 10 (1.1%) | 0 (0%) |  |
| II |  | 1,236 (45%) | 31 (38%) | 98 (26%) | 211 (23%) | 4 (11%) |  |
| III |  | 1,218 (44%) | 37 (46%) | 198 (53%) | 529 (56%) | 18 (51%) |  |
| IV |  | 242 (8.8%) | 12 (15%) | 76 (20%) | 187 (20%) | 13 (37%) |  |
| HR | 4,209 | 73 (15) | 69 (14) | 86 (21) | 88 (22) | 73 (13) | <0.001 |
| SBP | 4,221 | 126 (21) | 162 (29) | 125 (19) | 118 (21) | 135 (29) | <0.001 |
| Rales | 4,099 | 1,121 (42%) | 32 (41%) | 231 (63%) | 613 (66%) | 29 (85%) | <0.001 |
| Rales (type) | 3,040 |  |  |  |  |  |  |
| None |  | 1,211 (63%) | 27 (71%) | 112 (46%) | 334 (41%) | 15 (65%) |  |
| Single Base |  | 213 (11%) | 3 (7.9%) | 30 (12%) | 113 (14%) | 2 (8.7%) |  |
| Bibasilar |  | 501 (26%) | 8 (21%) | 100 (41%) | 365 (45%) | 6 (26%) |  |
| Edema | 3,643 | 1,013 (43%) | 55 (73%) | 321 (92%) | 790 (93%) | 32 (97%) | <0.001 |
| JVD | 3,230 | 352 (17%) | 23 (35%) | 144 (49%) | 454 (62%) | 31 (94%) | <0.001 |
| Hb | 3,989 | 13.29 (1.86) | 13.02 (1.73) | 12.51 (1.85) | 13.12 (2.09) | 11.75 (1.53) | <0.001 |
| eGFR | 4,248 | 66 (23) | 62 (22) | 60 (22) | 58 (24) | 48 (24) | <0.001 |
| NT-proBNP | 2,891 | 2,037 (708, 4,345) | 697 (257, 1,780) | 1,541 (710, 2,837) | 8,314 (4,145, 14,234) | 6,812 (4,956, 10,029) | <0.001 |
| LVEF | 3,811 | 35 (11) | 59 (5) | 54 (10) | 26 (8) | 59 (3) | <0.001 |
| HF type | 3,811 |  |  |  |  |  | <0.001 |
| HFmrEF |  | 414 (16%) | 3 (4.7%) | 71 (25%) | 28 (3.2%) | 3 (9.1%) |  |
| HFpEF |  | 198 (7.8%) | 61 (95%) | 170 (60%) | 2 (0.2%) | 30 (91%) |  |
| HFrEF |  | 1,938 (76%) | 0 (0%) | 40 (14%) | 853 (97%) | 0 (0%) |  |
| LVMi | 2,397 | 144 (58) | 122 (39) | 105 (34) | 145 (55) | 115 (41) | <0.001 |
| E/A | 1,331 | 1.00 (0.70, 1.52) | 0.90 (0.70, 1.30) | 0.99 (0.70, 1.40) | 2.27 (1.45, 3.00) | 1.00 (0.83, 1.00) | <0.001 |
| LAVi | 2,942 | 27.2 (3.6) | 25.6 (3.6) | 26.9 (4.4) | 28.6 (3.9) | 27.4 (3.2) | <0.001 |
| LVEDVi | 3,369 | 86 (16) | 67 (14) | 62 (11) | 90 (16) | 65 (12) | <0.001 |

1 Kruskal-Wallis rank sum test; Pearson’s Chi-squared test

Categorical variables presented as n(%), continuous variables as mean(SD) if distribution normal or Median (Q1, Q3) if distribution was non normal.

BMI: Body Mass Index; CAD: Coronary Artery Disease; AF: Atrial Fibrillation; COPD: Chronic Obstructive Pulmonary Disease; NYHA: New York Heart Association; HR: Heart Rate; SBP: Systolic Blood Pressure; JVD: Jugular Venous Distention; Hb: Hemoglobin; eGFR: estimated Glomerular Filtration Rate; BNP: Brain Natriuretic Peptide; LVEF: Left Ventricular Ejection Fraction; LVMi: Left Ventricular Mass index; LAVi: Left Atrial Volume index; LVEDVi: Left Ventricular End Diastolic Volume index

**Supplementary Figure 6S: Venn diagram of protein biomarkers significantly associated with the different phenotypes in BIOSTAT-CHF**

**
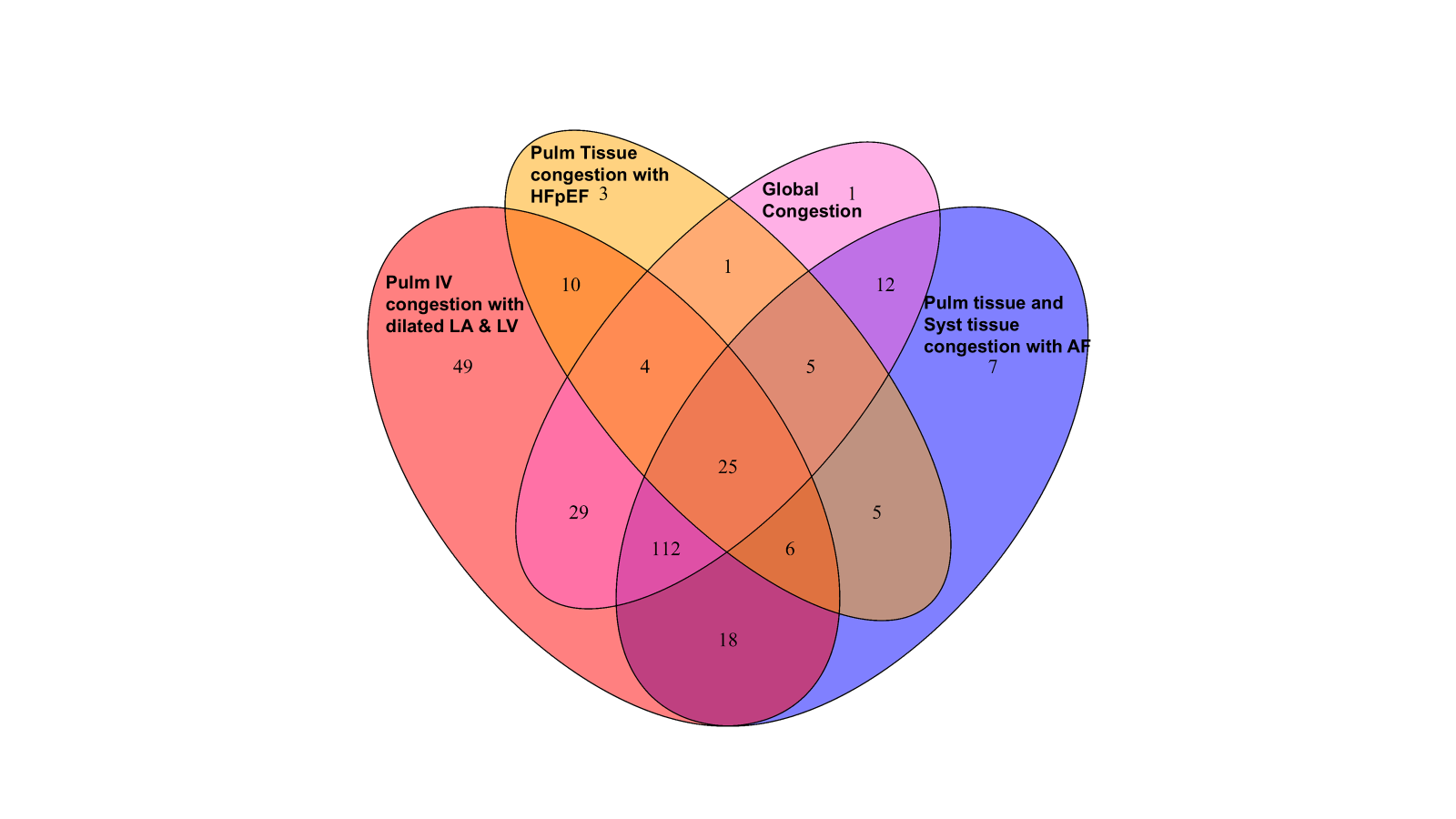
**

| **Phenotype** | **Total number of proteins associated with the phenotype** | **Biomarkers (OLINK Name)** |
| --- | --- | --- |
| **Pulm Tissue congestion with HFpEF** | 59 | REN, DECR1, DDX58 |
| **Pulm tissue and Syst tissue congestion with AF** | 190 | MPO, ITGA6, NF2, SPRY2, ICA1, S100A4, FURIN |
| **Pulm IV congestion with dilated LA & LV** | 253 | ITGB2, MMP_9, IL_6RA, CHIT1, EP_CAM, PCSK9, EGFR, RARRES2, SRC, IDUA, LOX_1, PIgR, SERPINA12, AMBP, IL16, IgG_Fc_receptor_IIb, GDF_2, MARCO, MMP_12, TGM2, CASA, PARP_1, HAOX1, ZBTB16, TPSAB1, IRAK1, TRIM5, DCTN1, TRAF2, TRIM21, EGLN1, NFATC3, EIF4G1, HSD11B1, HEXIM1, BACH1, PIK3AP1, DFFA, MASP1, CEACAM1, GPC1, ERBB2, MetAP 2, FASLG, Gal-1, ERBB4, S100A11, ICOSLG, ANXA1 |
| **Global congestion** | 190 | Gal_4 |

**Supplementary Figure 7S: Proportion of outcome for each phenotype stratified by a) age, b) sex, and c) LVEF category.**

| Figure a |
| --- |
| 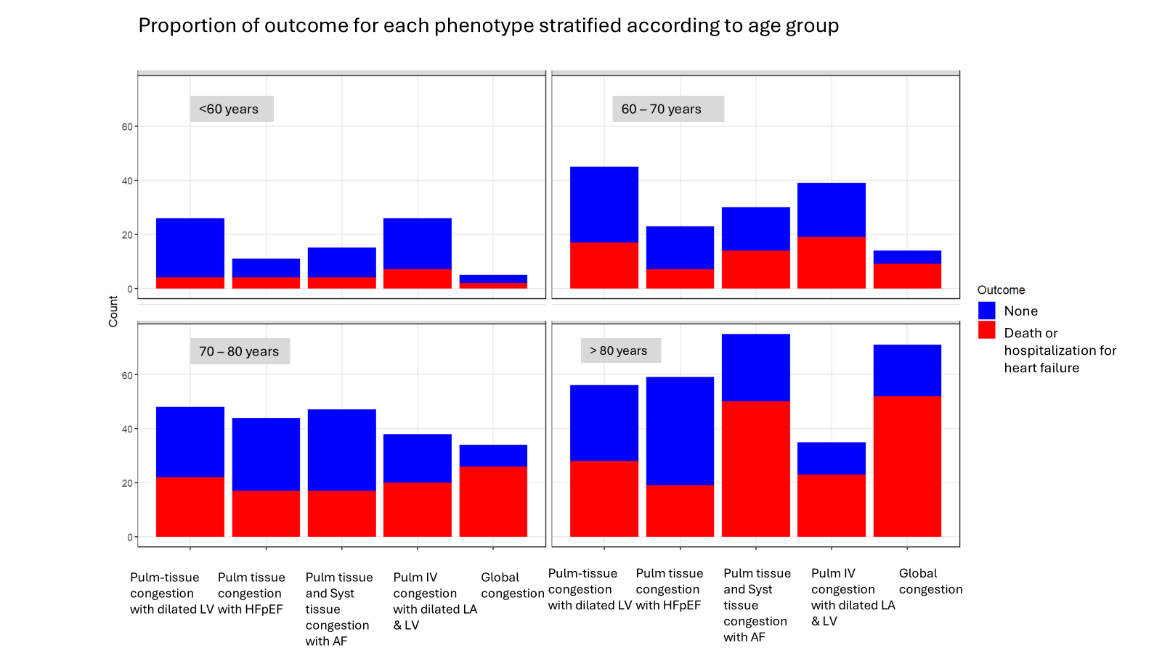 |
| Figure b |
| 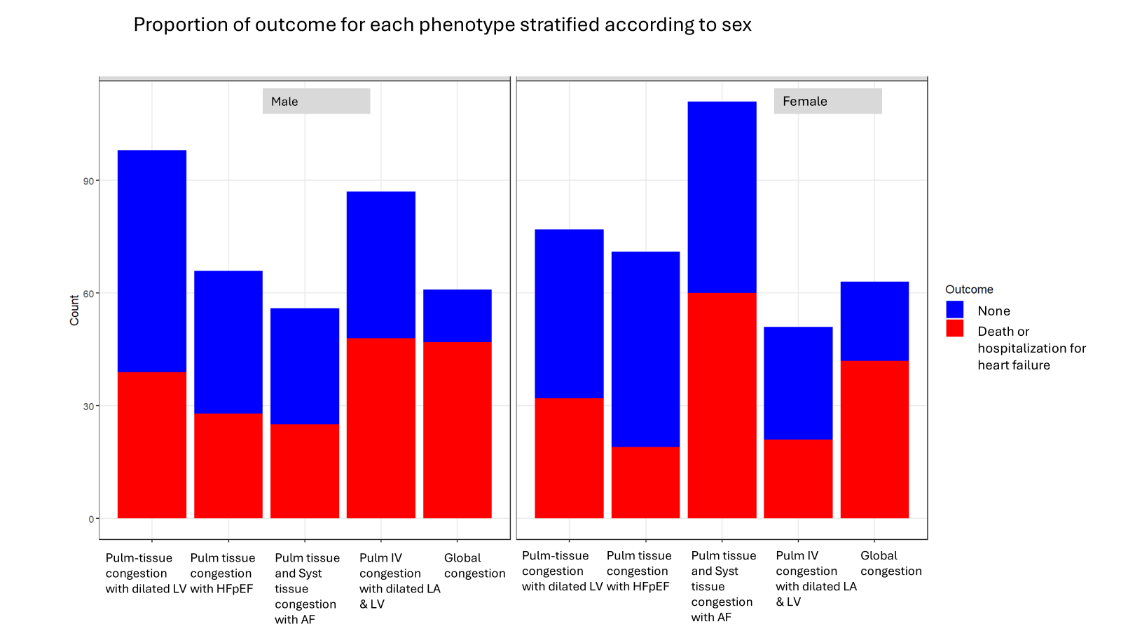 |
| Figure c |
| 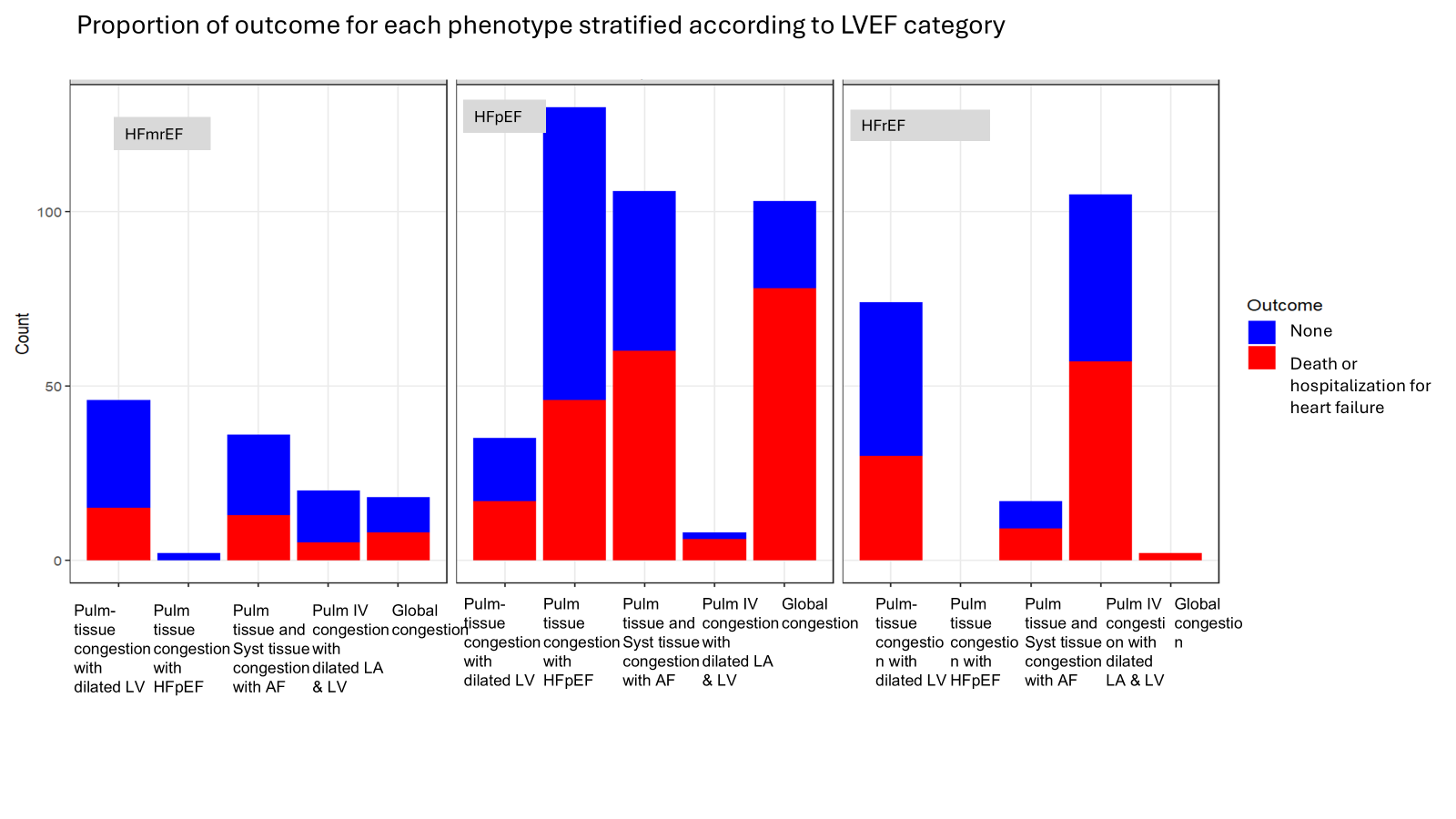 |

**Table 2S: Association of protein biomarkers with congestion phenotype using the multinomial logistic regression model.**

| **Biomarker** | **Pulm Tissue congestion with HFpEF** | | | **Pulm tissue and Syst tissue congestion with AF** | | | **Pulm IV congestion with dilated LA & LV** | | | **Global congestion** | | | P overall | P-  corrected |
| --- | --- | --- | --- | --- | --- | --- | --- | --- | --- | --- | --- | --- | --- | --- |
| (Olink name) | OR | CI | p | OR | CI | p | OR | CI | p | OR | CI | p |  |  |
| ITGB2 | 1.17 | 0.84, 1.62 | 0.3 | 1.02 | 0.87, 1.21 | 0.8 | 0.8 | 0.71, 0.89 | <0.001 | 0.92 | 0.56, 1.52 | 0.8 | <0.001 | 0.002 |
| MMP_9 | 0.95 | 0.76, 1.17 | 0.6 | 0.96 | 0.86, 1.07 | 0.4 | 1.13 | 1.04, 1.22 | 0.002 | 0.9 | 0.65, 1.25 | 0.5 | 0.014 | 0.019 |
| IL_6RA | 1.16 | 0.82, 1.65 | 0.4 | 1.16 | 0.97, 1.38 | 0.1 | 0.81 | 0.72, 0.90 | <0.001 | 1.36 | 0.80, 2.32 | 0.3 | <0.001 | <0.001 |
|  |  |  |  |  |  |  |  |  |  |  |  |  |  |  |
| CHIT1 | 1.05 | 0.89, 1.23 | 0.6 | 1.03 | 0.95, 1.11 | 0.5 | 0.89 | 0.84, 0.93 | <0.001 | 1.02 | 0.81, 1.28 | 0.9 | <0.001 | <0.001 |
| EP_CAM | 1.08 | 0.87, 1.33 | 0.5 | 0.99 | 0.89, 1.11 | 0.9 | 0.78 | 0.72, 0.85 | <0.001 | 0.97 | 0.71, 1.34 | 0.9 | <0.001 | <0.001 |
| PCSK9 | 1.15 | 0.78, 1.68 | 0.5 | 1.02 | 0.84, 1.24 | 0.8 | 0.73 | 0.63, 0.83 | <0.001 | 1.24 | 0.71, 2.19 | 0.5 | <0.001 | <0.001 |
| EGFR | 1.28 | 0.84, 1.95 | 0.3 | 0.88 | 0.70, 1.09 | 0.2 | 0.64 | 0.55, 0.75 | <0.001 | 0.78 | 0.41, 1.50 | 0.5 | <0.001 | <0.001 |
| RARRES2 | 1.04 | 0.69, 1.54 | 0.9 | 1.15 | 0.93, 1.41 | 0.2 | 0.76 | 0.67, 0.86 | <0.001 | 1.25 | 0.66, 2.38 | 0.5 | <0.001 | <0.001 |
| SRC | 0.98 | 0.82, 1.17 | 0.8 | 0.95 | 0.87, 1.04 | 0.2 | 0.9 | 0.84, 0.96 | <0.001 | 0.86 | 0.66, 1.13 | 0.3 | 0.018 | 0.024 |
| IDUA | 0.98 | 0.64, 1.51 | 0.9 | 0.81 | 0.66, 1.00 | 0.055 | 0.8 | 0.69, 0.93 | 0.003 | 0.65 | 0.35, 1.18 | 0.2 | 0.016 | 0.021 |
| LOX_1 | 0.73 | 0.52, 1.03 | 0.074 | 0.93 | 0.79, 1.10 | 0.4 | 1.39 | 1.24, 1.55 | <0.001 | 0.94 | 0.57, 1.54 | 0.8 | <0.001 | <0.001 |
| PIgR | 2.39 | 0.43, 13.2 | 0.3 | 1.15 | 0.50, 2.64 | 0.7 | 3.71 | 2.02, 6.80 | <0.001 | 11.4 | 0.90, 146 | 0.061 | <0.001 | <0.001 |
| SERPINA12 | 0.83 | 0.66, 1.04 | 0.1 | 1.04 | 0.93, 1.15 | 0.5 | 1.14 | 1.06, 1.23 | <0.001 | 1.14 | 0.84, 1.55 | 0.4 | 0.002 | 0.003 |
| AMBP | 2.01 | 0.96, 4.23 | 0.065 | 0.98 | 0.68, 1.40 | 0.9 | 0.48 | 0.37, 0.61 | <0.001 | 1.97 | 0.64, 6.00 | 0.2 | <0.001 | <0.001 |
| IL16 | 1.26 | 0.89, 1.79 | 0.2 | 1.17 | 0.98, 1.39 | 0.08 | 1.25 | 1.10, 1.41 | <0.001 | 1.61 | 0.98, 2.67 | 0.062 | 0.002 | 0.003 |
| IgG Fc receptor IIb | 1.13 | 0.91, 1.42 | 0.3 | 1.12 | 1.00, 1.25 | 0.053 | 1.1 | 1.02, 1.19 | 0.018 | 1.4 | 0.98, 2.00 | 0.064 | 0.019 | 0.025 |
| GDF_2 | 1 | 0.74, 1.36 | 0.9 | 0.95 | 0.81, 1.10 | 0.5 | 0.84 | 0.76, 0.94 | 0.002 | 1.04 | 0.66, 1.64 | 0.9 | 0.037 | 0.047 |
| MARCO | 1.29 | 0.53, 3.15 | 0.6 | 1.02 | 0.65, 1.59 | 0.9 | 0.44 | 0.33, 0.60 | <0.001 | 1.02 | 0.27, 3.90 | 0.9 | <0.001 | <0.001 |
| MMP_12 | 0.96 | 0.75, 1.22 | 0.7 | 0.93 | 0.82, 1.05 | 0.2 | 0.85 | 0.78, 0.92 | <0.001 | 1.21 | 0.84, 1.74 | 0.3 | 0.003 | 0.004 |
| TGM2 | 0.82 | 0.61, 1.10 | 0.2 | 0.91 | 0.78, 1.06 | 0.2 | 1.37 | 1.22, 1.54 | <0.001 | 1.02 | 0.64, 1.62 | 0.9 | <0.001 | <0.001 |
| CASA | 0.89 | 0.71, 1.12 | 0.3 | 1.1 | 0.99, 1.21 | 0.064 | 1.23 | 1.15, 1.31 | <0.001 | 1.25 | 0.96, 1.63 | 0.1 | <0.001 | <0.001 |
| PARP_1 | 0.77 | 0.56, 1.06 | 0.11 | 0.96 | 0.84, 1.11 | 0.6 | 1.18 | 1.09, 1.29 | <0.001 | 0.95 | 0.62, 1.46 | 0.8 | <0.001 | <0.001 |
| HAOX1 | 0.87 | 0.75, 1.00 | 0.058 | 0.99 | 0.93, 1.06 | 0.9 | 1.11 | 1.06, 1.16 | <0.001 | 0.9 | 0.73, 1.11 | 0.3 | <0.001 | <0.001 |
| ZBTB16 | 0.77 | 0.58, 1.03 | 0.083 | 0.9 | 0.79, 1.03 | 0.11 | 1.16 | 1.06, 1.25 | <0.001 | 0.89 | 0.59, 1.33 | 0.6 | <0.001 | <0.001 |
| TPSAB1 | 1.45 | 0.99, 2.12 | 0.057 | 1.04 | 0.86, 1.25 | 0.7 | 0.87 | 0.76, 0.99 | 0.036 | 0.64 | 0.37, 1.10 | 0.11 | 0.021 | 0.027 |
| IRAK1 | 0.77 | 0.52, 1.14 | 0.2 | 0.88 | 0.73, 1.06 | 0.2 | 1.21 | 1.08, 1.36 | <0.001 | 1.16 | 0.72, 1.88 | 0.5 | 0.002 | 0.003 |
| TRIM5 | 0.69 | 0.44, 1.10 | 0.12 | 0.94 | 0.76, 1.15 | 0.6 | 1.36 | 1.20, 1.55 | <0.001 | 1.05 | 0.58, 1.90 | 0.9 | <0.001 | <0.001 |
| DCTN1 | 0.85 | 0.67, 1.07 | 0.2 | 0.9 | 0.80, 1.01 | 0.075 | 1.13 | 1.04, 1.22 | 0.002 | 0.87 | 0.61, 1.23 | 0.4 | <0.001 | 0.002 |
| TRAF2 | 0.67 | 0.44, 1.03 | 0.07 | 0.98 | 0.81, 1.18 | 0.8 | 1.19 | 1.05, 1.35 | 0.006 | 0.78 | 0.42, 1.43 | 0.4 | 0.01 | 0.014 |
| TRIM21 | 0.84 | 0.62, 1.14 | 0.3 | 0.86 | 0.74, 1.00 | 0.053 | 1.17 | 1.06, 1.29 | <0.001 | 0.78 | 0.49, 1.24 | 0.3 | <0.001 | <0.001 |
| EGLN1 | 0.9 | 0.52, 1.54 | 0.7 | 1.1 | 0.86, 1.41 | 0.4 | 1.88 | 1.62, 2.18 | <0.001 | 1.17 | 0.58, 2.38 | 0.7 | <0.001 | <0.001 |
| NFATC3 | 0.93 | 0.66, 1.30 | 0.7 | 0.95 | 0.80, 1.12 | 0.5 | 1.26 | 1.14, 1.38 | <0.001 | 1.02 | 0.64, 1.62 | 0.9 | <0.001 | <0.001 |
| EIF4G1 | 0.86 | 0.71, 1.03 | 0.1 | 0.94 | 0.86, 1.03 | 0.2 | 1.09 | 1.03, 1.16 | 0.005 | 1.01 | 0.77, 1.32 | 0.9 | 0.006 | 0.008 |
| HSD11B1 | 0.68 | 0.42, 1.09 | 0.11 | 0.96 | 0.76, 1.22 | 0.7 | 1.79 | 1.51, 2.11 | <0.001 | 1.17 | 0.57, 2.37 | 0.7 | <0.001 | <0.001 |
| HEXIM1 | 0.85 | 0.67, 1.08 | 0.2 | 0.96 | 0.86, 1.07 | 0.5 | 1.14 | 1.05, 1.23 | <0.001 | 1.04 | 0.75, 1.45 | 0.8 | 0.004 | 0.006 |
| BACH1 | 1.01 | 0.66, 1.54 | 0.9 | 1.01 | 0.82, 1.25 | 0.9 | 1.27 | 1.11, 1.46 | <0.001 | 0.89 | 0.46, 1.73 | 0.7 | 0.015 | 0.021 |
| PIK3AP1 | 0.8 | 0.60, 1.07 | 0.13 | 0.96 | 0.84, 1.10 | 0.6 | 1.16 | 1.06, 1.27 | <0.001 | 0.88 | 0.58, 1.34 | 0.6 | 0.004 | 0.006 |
| DFFA | 0.78 | 0.57, 1.05 | 0.1 | 0.96 | 0.84, 1.10 | 0.5 | 1.22 | 1.12, 1.34 | <0.001 | 1.01 | 0.68, 1.51 | 0.9 | <0.001 | <0.001 |
| MASP1 | 0.94 | 0.49, 1.79 | 0.9 | 1.26 | 0.91, 1.75 | 0.2 | 1.77 | 1.41, 2.23 | <0.001 | 0.78 | 0.30, 2.05 | 0.6 | <0.001 | <0.001 |
| CEACAM1 | 0.42 | 0.17, 1.03 | 0.057 | 1.33 | 0.76, 2.33 | 0.3 | 3.76 | 2.45, 5.75 | <0.001 | 1.79 | 0.32, 10.0 | 0.5 | <0.001 | <0.001 |
| GPC1 | 1.31 | 0.81, 2.12 | 0.3 | 1 | 0.79, 1.28 | 0.9 | 1.49 | 1.25, 1.76 | <0.001 | 1.68 | 0.82, 3.41 | 0.2 | <0.001 | <0.001 |
| ERBB2 | 0.76 | 0.44, 1.29 | 0.3 | 1.02 | 0.78, 1.35 | 0.9 | 1.42 | 1.16, 1.73 | <0.001 | 0.95 | 0.42, 2.16 | 0.9 | 0.006 | 0.009 |
| MetAP 2 | 0.85 | 0.62, 1.15 | 0.3 | 0.96 | 0.82, 1.12 | 0.6 | 1.18 | 1.07, 1.32 | 0.002 | 0.97 | 0.61, 1.53 | 0.9 | 0.011 | 0.015 |
| FASLG | 1.38 | 0.92, 2.06 | 0.12 | 1.15 | 0.94, 1.41 | 0.2 | 0.86 | 0.75, 0.98 | 0.029 | 0.86 | 0.48, 1.54 | 0.6 | 0.024 | 0.031 |
| Gal-1 | 1.71 | 0.80, 3.66 | 0.2 | 1.2 | 0.79, 1.81 | 0.4 | 0.69 | 0.52, 0.92 | 0.012 | 1.1 | 0.32, 3.80 | 0.9 | 0.035 | 0.045 |
| ERBB4 | 0.73 | 0.41, 1.32 | 0.3 | 1.01 | 0.74, 1.36 | 0.9 | 1.82 | 1.46, 2.27 | <0.001 | 1.06 | 0.42, 2.63 | 0.9 | <0.001 | <0.001 |
| S100A11 | 0.78 | 0.39, 1.54 | 0.5 | 1.26 | 0.93, 1.70 | 0.14 | 1.71 | 1.40, 2.10 | <0.001 | 2.03 | 0.99, 4.15 | 0.054 | <0.001 | <0.001 |
| ICOSLG | 1.25 | 0.68, 2.29 | 0.5 | 0.82 | 0.60, 1.11 | 0.2 | 0.72 | 0.57, 0.89 | 0.003 | 1.47 | 0.60, 3.56 | 0.4 | 0.02 | 0.026 |
| ANXA1 | 0.81 | 0.55, 1.20 | 0.3 | 1.03 | 0.86, 1.22 | 0.8 | 1.28 | 1.14, 1.43 | <0.001 | 1.17 | 0.72, 1.88 | 0.5 | <0.001 | <0.001 |
| MPO | 1.22 | 0.88, 1.69 | 0.2 | 1.21 | 1.03, 1.43 | 0.022 | 0.92 | 0.82, 1.03 | 0.2 | 1.2 | 0.73, 1.96 | 0.5 | 0.035 | 0.045 |
| ITGA6 | 0.83 | 0.61, 1.13 | 0.2 | 0.71 | 0.60, 0.84 | <0.001 | 1.03 | 0.94, 1.13 | 0.5 | 0.81 | 0.51, 1.30 | 0.4 | <0.001 | <0.001 |
| NF2 | 0.85 | 0.69, 1.04 | 0.11 | 0.87 | 0.79, 0.96 | 0.007 | 1.02 | 0.95, 1.08 | 0.6 | 0.79 | 0.57, 1.09 | 0.2 | 0.009 | 0.013 |
| SPRY2 | 0.82 | 0.66, 1.01 | 0.065 | 0.89 | 0.81, 0.99 | 0.029 | 1.04 | 0.97, 1.10 | 0.3 | 0.88 | 0.65, 1.20 | 0.4 | 0.022 | 0.029 |
| ICA1 | 0.65 | 0.40, 1.06 | 0.084 | 0.76 | 0.60, 0.94 | 0.013 | 1.04 | 0.91, 1.18 | 0.6 | 0.76 | 0.38, 1.48 | 0.4 | 0.021 | 0.027 |
| S100A4 | 0.85 | 0.56, 1.29 | 0.4 | 0.69 | 0.56, 0.85 | <0.001 | 1 | 0.86, 1.15 | 0.9 | 0.66 | 0.35, 1.24 | 0.2 | 0.006 | 0.008 |
| FURIN | 0.97 | 0.61, 1.54 | 0.9 | 1.28 | 1.01, 1.62 | 0.039 | 0.89 | 0.75, 1.05 | 0.2 | 0.57 | 0.29, 1.11 | 0.1 | 0.037 | 0.046 |
| REN | 0.74 | 0.60, 0.91 | 0.004 | 1 | 0.89, 1.12 | 0.9 | 1.04 | 0.96, 1.13 | 0.3 | 0.8 | 0.58, 1.09 | 0.2 | 0.025 | 0.032 |
| DECR1 | 0.8 | 0.66, 0.97 | 0.02 | 0.92 | 0.85, 1.01 | 0.082 | 1.06 | 1.00, 1.12 | 0.064 | 1.03 | 0.80, 1.33 | 0.8 | 0.007 | 0.01 |
| DDX58 | 0.67 | 0.46, 0.97 | 0.035 | 0.92 | 0.78, 1.07 | 0.3 | 1.1 | 1.00, 1.22 | 0.055 | 0.68 | 0.39, 1.18 | 0.2 | 0.01 | 0.013 |
| gal_4 | 1.2 | 0.92, 1.56 | 0.2 | 1.13 | 0.98, 1.29 | 0.083 | 1.01 | 0.92, 1.10 | 0.9 | 1.65 | 1.12, 2.41 | 0.011 | 0.032 | 0.041 |
| pon3 | 1.26 | 1.00, 1.58 | 0.051 | 0.86 | 0.77, 0.95 | 0.005 | 0.64 | 0.59, 0.69 | <0.001 | 0.85 | 0.62, 1.18 | 0.3 | <0.001 | <0.001 |
| CD40_ligand | 0.87 | 0.72, 1.05 | 0.2 | 0.9 | 0.82, 0.98 | 0.022 | 0.92 | 0.87, 0.99 | 0.019 | 0.84 | 0.62, 1.12 | 0.2 | 0.018 | 0.024 |
| IL-1ra | 1.06 | 0.82, 1.38 | 0.6 | 1.42 | 1.25, 1.61 | <0.001 | 1.22 | 1.12, 1.34 | <0.001 | 1.1 | 0.74, 1.62 | 0.6 | <0.001 | <0.001 |
| TIE2 | 0.86 | 0.45, 1.63 | 0.6 | 1.6 | 1.14, 2.26 | 0.007 | 2.62 | 2.04, 3.37 | <0.001 | 1.92 | 0.67, 5.46 | 0.2 | <0.001 | <0.001 |
| IL1RL2 | 1.12 | 0.71, 1.77 | 0.6 | 1.46 | 1.16, 1.83 | <0.001 | 0.8 | 0.68, 0.94 | 0.006 | 0.83 | 0.42, 1.64 | 0.6 | <0.001 | <0.001 |
| GIF | 0.98 | 0.82, 1.17 | 0.8 | 0.9 | 0.82, 0.98 | 0.013 | 0.87 | 0.82, 0.92 | <0.001 | 0.92 | 0.71, 1.19 | 0.5 | <0.001 | <0.001 |
| CNTNAP2 | 1.11 | 0.72, 1.70 | 0.6 | 1.34 | 1.09, 1.64 | 0.005 | 1.19 | 1.02, 1.38 | 0.026 | 1.41 | 0.79, 2.51 | 0.2 | 0.023 | 0.03 |
| EDAR | 0.9 | 0.62, 1.29 | 0.6 | 0.69 | 0.56, 0.84 | <0.001 | 0.78 | 0.69, 0.90 | <0.001 | 0.73 | 0.41, 1.33 | 0.3 | <0.001 | <0.001 |
| GALNT3 | 0.77 | 0.48, 1.23 | 0.3 | 1.33 | 1.07, 1.65 | 0.009 | 1.65 | 1.42, 1.91 | <0.001 | 1.69 | 0.93, 3.07 | 0.084 | <0.001 | <0.001 |
| HNMT | 1.05 | 0.77, 1.42 | 0.8 | 1.3 | 1.12, 1.51 | <0.001 | 1.47 | 1.33, 1.63 | <0.001 | 1.45 | 0.96, 2.22 | 0.081 | <0.001 | <0.001 |
| KLRD1 | 1.15 | 0.83, 1.58 | 0.4 | 1.33 | 1.13, 1.57 | <0.001 | 1.14 | 1.01, 1.27 | 0.028 | 1.56 | 0.97, 2.52 | 0.069 | 0.002 | 0.002 |
| STC1 | 0.93 | 0.59, 1.45 | 0.7 | 1.94 | 1.48, 2.53 | <0.001 | 1.76 | 1.46, 2.12 | <0.001 | 1.23 | 0.58, 2.61 | 0.6 | <0.001 | <0.001 |
| KLK13 | 0.77 | 0.50, 1.19 | 0.2 | 0.68 | 0.55, 0.85 | <0.001 | 0.84 | 0.73, 0.98 | 0.027 | 1.18 | 0.64, 2.18 | 0.6 | 0.003 | 0.005 |
| EGF | 0.91 | 0.78, 1.05 | 0.2 | 0.92 | 0.85, 0.99 | 0.018 | 0.94 | 0.90, 0.99 | 0.024 | 0.82 | 0.66, 1.03 | 0.088 | 0.013 | 0.017 |
| MIA | 0.67 | 0.36, 1.25 | 0.2 | 0.63 | 0.46, 0.86 | 0.003 | 0.71 | 0.56, 0.89 | 0.003 | 0.58 | 0.23, 1.42 | 0.2 | 0.003 | 0.004 |
| CD207 | 1.3 | 0.84, 2.00 | 0.2 | 0.79 | 0.63, 0.99 | 0.038 | 0.79 | 0.67, 0.92 | 0.002 | 0.85 | 0.44, 1.65 | 0.6 | 0.007 | 0.01 |
| ADAM-TS 15 | 1.24 | 0.86, 1.78 | 0.3 | 1.49 | 1.23, 1.79 | <0.001 | 1.15 | 1.01, 1.31 | 0.028 | 1.09 | 0.64, 1.86 | 0.8 | <0.001 | <0.001 |
| CEACAM5 | 1.1 | 0.84, 1.44 | 0.5 | 1.28 | 1.12, 1.45 | <0.001 | 1.22 | 1.11, 1.34 | <0.001 | 1.36 | 0.95, 1.93 | 0.091 | <0.001 | <0.001 |
| PAI | 0.69 | 0.57, 0.84 | <0.001 | 1.01 | 0.92, 1.11 | 0.8 | 1.12 | 1.05, 1.19 | <0.001 | 0.79 | 0.59, 1.05 | 0.1 | <0.001 | <0.001 |
| TR_AP | 1.46 | 1.08, 1.96 | 0.012 | 0.94 | 0.81, 1.10 | 0.5 | 0.53 | 0.47, 0.59 | <0.001 | 0.97 | 0.61, 1.53 | 0.9 | <0.001 | <0.001 |
| CCL22 | 1.22 | 1.03, 1.45 | 0.024 | 1.09 | 0.99, 1.20 | 0.08 | 0.78 | 0.72, 0.85 | <0.001 | 0.91 | 0.65, 1.27 | 0.6 | <0.001 | <0.001 |
| SORT1 | 0.53 | 0.32, 0.85 | 0.009 | 1.34 | 0.97, 1.86 | 0.072 | 1.59 | 1.27, 2.01 | <0.001 | 1 | 0.39, 2.56 | 0.9 | <0.001 | <0.001 |
| PSIP1 | 0.71 | 0.50, 0.99 | 0.041 | 1.01 | 0.88, 1.15 | 0.9 | 1.34 | 1.24, 1.45 | <0.001 | 1.2 | 0.86, 1.68 | 0.3 | <0.001 | <0.001 |
| CLEC4A | 1.88 | 1.15, 3.07 | 0.012 | 0.96 | 0.75, 1.23 | 0.7 | 0.7 | 0.59, 0.84 | <0.001 | 0.74 | 0.35, 1.56 | 0.4 | <0.001 | <0.001 |
| PRDX3 | 0.7 | 0.49, 0.99 | 0.046 | 0.91 | 0.78, 1.07 | 0.2 | 1.16 | 1.05, 1.28 | 0.004 | 0.9 | 0.56, 1.45 | 0.7 | 0.002 | 0.003 |
| PADI2 | 0.41 | 0.21, 0.79 | 0.007 | 0.9 | 0.72, 1.12 | 0.4 | 1.26 | 1.11, 1.43 | <0.001 | 1.34 | 0.84, 2.14 | 0.2 | <0.001 | <0.001 |
| ITGB6 | 0.58 | 0.35, 0.96 | 0.033 | 0.82 | 0.64, 1.06 | 0.13 | 1.53 | 1.28, 1.83 | <0.001 | 1.98 | 0.96, 4.06 | 0.063 | <0.001 | <0.001 |
| CPE | 0.51 | 0.32, 0.81 | 0.004 | 1 | 0.79, 1.28 | 0.9 | 1.73 | 1.45, 2.06 | <0.001 | 1.53 | 0.75, 3.15 | 0.2 | <0.001 | <0.001 |
| LDL_RECEPTOR | 1.25 | 0.97, 1.60 | 0.079 | 0.95 | 0.83, 1.08 | 0.4 | 0.61 | 0.56, 0.67 | <0.001 | 0.68 | 0.46, 1.00 | 0.049 | <0.001 | <0.001 |
| MEPE | 1.32 | 1.00, 1.75 | 0.052 | 0.89 | 0.77, 1.03 | 0.12 | 0.82 | 0.74, 0.91 | <0.001 | 1.6 | 1.06, 2.42 | 0.024 | <0.001 | <0.001 |
| CPA1 | 0.99 | 0.81, 1.21 | 0.9 | 1.05 | 0.95, 1.16 | 0.3 | 1.09 | 1.02, 1.17 | 0.013 | 1.39 | 1.04, 1.85 | 0.027 | 0.03 | 0.038 |
| JAM_A | 0.83 | 0.66, 1.04 | 0.1 | 0.98 | 0.88, 1.09 | 0.7 | 1.1 | 1.03, 1.19 | 0.007 | 1.34 | 1.02, 1.75 | 0.034 | 0.004 | 0.006 |
| SLAMF7 | 0.82 | 0.61, 1.10 | 0.2 | 1.05 | 0.91, 1.22 | 0.5 | 1.43 | 1.29, 1.58 | <0.001 | 1.84 | 1.27, 2.67 | <0.001 | <0.001 | <0.001 |
| Brother of CDO | 0.72 | 0.40, 1.30 | 0.3 | 1.07 | 0.80, 1.43 | 0.7 | 3.46 | 2.80, 4.26 | <0.001 | 4.14 | 1.83, 9.39 | <0.001 | <0.001 | <0.001 |
| RAGE | 0.97 | 0.65, 1.43 | 0.9 | 1.01 | 0.83, 1.23 | 0.9 | 1.99 | 1.71, 2.32 | <0.001 | 3.86 | 1.89, 7.86 | <0.001 | <0.001 | <0.001 |
| CD84 | 0.88 | 0.55, 1.41 | 0.6 | 1.21 | 0.96, 1.53 | 0.1 | 1.46 | 1.24, 1.72 | <0.001 | 2.09 | 1.06, 4.11 | 0.034 | <0.001 | <0.001 |
| VSIG2 | 1.11 | 0.83, 1.49 | 0.5 | 1.11 | 0.96, 1.28 | 0.2 | 1.31 | 1.18, 1.45 | <0.001 | 2.01 | 1.34, 3.01 | <0.001 | <0.001 | <0.001 |
| CEACAM8 | 0.87 | 0.64, 1.18 | 0.4 | 1.15 | 1.00, 1.33 | 0.055 | 1.61 | 1.46, 1.78 | <0.001 | 1.86 | 1.26, 2.73 | 0.002 | <0.001 | <0.001 |
| Dkk1 | 0.77 | 0.58, 1.03 | 0.081 | 1.03 | 0.89, 1.19 | 0.7 | 1.33 | 1.20, 1.47 | <0.001 | 2.08 | 1.39, 3.10 | <0.001 | <0.001 | <0.001 |
| GT | 1.08 | 0.81, 1.45 | 0.6 | 1.07 | 0.92, 1.23 | 0.4 | 1.12 | 1.01, 1.25 | 0.025 | 1.81 | 1.34, 2.45 | <0.001 | 0.004 | 0.006 |
| ACE2 | 0.73 | 0.53, 1.00 | 0.052 | 1.12 | 0.96, 1.31 | 0.14 | 1.73 | 1.55, 1.92 | <0.001 | 1.96 | 1.29, 2.98 | 0.002 | <0.001 | <0.001 |
| TNFRSF13B | 0.78 | 0.53, 1.16 | 0.2 | 1.04 | 0.86, 1.25 | 0.7 | 1.37 | 1.20, 1.56 | <0.001 | 2.38 | 1.62, 3.51 | <0.001 | <0.001 | <0.001 |
| HSP 27 | 1.12 | 0.71, 1.78 | 0.6 | 1.15 | 0.91, 1.46 | 0.2 | 1.29 | 1.09, 1.52 | 0.003 | 2.61 | 1.08, 6.30 | 0.033 | 0.006 | 0.009 |
| VEGFD | 0.79 | 0.53, 1.17 | 0.2 | 0.91 | 0.73, 1.13 | 0.4 | 2.6 | 2.14, 3.17 | <0.001 | 3.19 | 1.34, 7.56 | 0.008 | <0.001 | <0.001 |
| CLEC4C | 1.15 | 0.82, 1.62 | 0.4 | 0.93 | 0.78, 1.10 | 0.4 | 0.69 | 0.61, 0.77 | <0.001 | 0.52 | 0.32, 0.85 | 0.009 | <0.001 | <0.001 |
| CXCL12 | 0.83 | 0.31, 2.20 | 0.7 | 1.22 | 0.76, 1.98 | 0.4 | 2.33 | 1.67, 3.26 | <0.001 | 4.42 | 1.31, 14.9 | 0.016 | <0.001 | <0.001 |
| LAMP3 | 0.89 | 0.64, 1.23 | 0.5 | 1.1 | 0.94, 1.29 | 0.3 | 1.15 | 1.03, 1.29 | 0.015 | 2.21 | 1.36, 3.59 | <0.001 | 0.002 | 0.003 |
| MSLN | 1.09 | 0.82, 1.45 | 0.5 | 1.15 | 1.0, 1.32 | 0.059 | 1.35 | 1.23, 1.49 | <0.001 | 2.31 | 1.56, 3.43 | <0.001 | <0.001 | <0.001 |
| IGF1R | 0.86 | 0.50, 1.49 | 0.6 | 1.3 | 1.00, 1.69 | 0.05 | 3.04 | 2.53, 3.64 | <0.001 | 6.23 | 3.60, 10.8 | <0.001 | <0.001 | <0.001 |
| CYR61 | 1.07 | 0.77, 1.50 | 0.7 | 0.98 | 0.83, 1.16 | 0.8 | 1.48 | 1.32, 1.67 | <0.001 | 1.78 | 1.10, 2.88 | 0.02 | <0.001 | <0.001 |
| PVRL4 | 1.19 | 0.85, 1.65 | 0.3 | 1.13 | 0.96, 1.34 | 0.14 | 1.27 | 1.13, 1.43 | <0.001 | 2.71 | 1.76, 4.16 | <0.001 | <0.001 | <0.001 |
| ABL1 | 0.86 | 0.65, 1.14 | 0.3 | 1.05 | 0.92, 1.19 | 0.5 | 1.25 | 1.15, 1.36 | <0.001 | 1.44 | 1.05, 1.97 | 0.022 | <0.001 | <0.001 |
| RET | 0.9 | 0.64, 1.28 | 0.6 | 0.95 | 0.80, 1.14 | 0.6 | 0.52 | 0.46, 0.59 | <0.001 | 0.41 | 0.24, 0.68 | <0.001 | <0.001 | <0.001 |
| CRNN | 1.12 | 0.89, 1.39 | 0.3 | 1.11 | 0.99, 1.24 | 0.075 | 0.87 | 0.80, 0.94 | <0.001 | 1.4 | 1.04, 1.87 | 0.025 | <0.001 | <0.001 |
| CD160 | 1.29 | 0.95, 1.74 | 0.1 | 1.06 | 0.91, 1.24 | 0.5 | 1.12 | 1.01, 1.25 | 0.04 | 1.61 | 1.04, 2.49 | 0.033 | 0.04 | 0.05 |
| TNFRSF4 | 1.06 | 0.77, 1.47 | 0.7 | 1.12 | 0.95, 1.32 | 0.2 | 1.21 | 1.08, 1.36 | <0.001 | 2.2 | 1.44, 3.38 | <0.001 | <0.001 | <0.001 |
| MAD homolog 5 | 1.13 | 0.48, 2.65 | 0.8 | 0.61 | 0.32, 1.16 | 0.13 | 0.3 | 0.19, 0.47 | <0.001 | 0.19 | 0.04, 0.90 | 0.036 | <0.001 | <0.001 |
| FR-alpha | 1.22 | 0.86, 1.73 | 0.3 | 1.16 | 0.98, 1.39 | 0.09 | 1.31 | 1.16, 1.48 | <0.001 | 2.88 | 1.79, 4.63 | <0.001 | <0.001 | <0.001 |
| casp_3 | 0.8 | 0.68, 0.94 | 0.008 | 0.89 | 0.83, 0.97 | 0.005 | 0.99 | 0.94, 1.05 | 0.8 | 0.82 | 0.64, 1.05 | 0.11 | 0.002 | 0.003 |
| MMP_7 | 1.47 | 1.02, 2.11 | 0.039 | 1.22 | 1.03, 1.45 | 0.022 | 0.95 | 0.85, 1.05 | 0.3 | 1.51 | 0.87, 2.63 | 0.14 | 0.005 | 0.007 |
| HB_EGF | 0.59 | 0.36, 0.95 | 0.029 | 0.73 | 0.59, 0.91 | 0.005 | 0.99 | 0.86, 1.14 | 0.9 | 0.6 | 0.29, 1.22 | 0.2 | 0.004 | 0.006 |
| GLB1 | 0.56 | 0.35, 0.91 | 0.019 | 0.77 | 0.61, 0.96 | 0.019 | 1.07 | 0.94, 1.23 | 0.3 | 0.76 | 0.39, 1.48 | 0.4 | 0.006 | 0.008 |
| FADD | 0.74 | 0.56, 0.99 | 0.041 | 0.86 | 0.75, 0.98 | 0.021 | 1.06 | 0.98, 1.15 | 0.2 | 0.93 | 0.64, 1.36 | 0.7 | 0.008 | 0.011 |
| RETN | 1.11 | 0.84, 1.46 | 0.5 | 1.23 | 1.08, 1.41 | 0.003 | 1.07 | 0.98, 1.18 | 0.14 | 2.32 | 1.59, 3.38 | <0.001 | <0.001 | <0.001 |
| PSP_D | 0.97 | 0.75, 1.25 | 0.8 | 0.85 | 0.74, 0.97 | 0.015 | 0.97 | 0.88, 1.06 | 0.5 | 1.51 | 1.07, 2.15 | 0.021 | 0.019 | 0.025 |
| FAS | 1.33 | 0.97, 1.81 | 0.074 | 1.23 | 1.05, 1.44 | 0.009 | 0.96 | 0.86, 1.08 | 0.5 | 2.19 | 1.47, 3.25 | <0.001 | <0.001 | <0.001 |
| PRTN3 | 1.3 | 0.99, 1.69 | 0.056 | 1.21 | 1.06, 1.39 | 0.006 | 1.08 | 0.97, 1.19 | 0.15 | 1.56 | 1.09, 2.23 | 0.016 | 0.005 | 0.007 |
| SHPS_1 | 1.32 | 0.98, 1.78 | 0.072 | 1.31 | 1.13, 1.52 | <0.001 | 1.1 | 0.99, 1.23 | 0.065 | 2.58 | 1.72, 3.85 | <0.001 | <0.001 | <0.001 |
| SCGB3A2 | 1.09 | 0.91, 1.30 | 0.4 | 1.22 | 1.13, 1.33 | <0.001 | 1.06 | 0.99, 1.13 | 0.093 | 1.46 | 1.20, 1.77 | <0.001 | <0.001 | <0.001 |
| CD93 | 1.25 | 0.89, 1.77 | 0.2 | 1.19 | 1.00, 1.41 | 0.044 | 1.11 | 0.99, 1.24 | 0.083 | 3.27 | 1.82, 5.87 | <0.001 | <0.001 | <0.001 |
| ICAM_2 | 1.29 | 0.95, 1.75 | 0.1 | 1.31 | 1.13, 1.53 | <0.001 | 1.09 | 0.98, 1.21 | 0.11 | 2.46 | 1.60, 3.79 | <0.001 | <0.001 | <0.001 |
| LPL | 1.13 | 0.73, 1.75 | 0.6 | 1.8 | 1.43, 2.28 | <0.001 | 1.12 | 0.96, 1.30 | 0.2 | 2.8 | 1.33, 5.89 | 0.007 | <0.001 | <0.001 |
| PRKCQ | 1.34 | 0.94, 1.91 | 0.11 | 0.78 | 0.62, 0.98 | 0.033 | 0.95 | 0.82, 1.10 | 0.5 | 0.39 | 0.17, 0.87 | 0.022 | 0.01 | 0.014 |
| SIT1 | 0.93 | 0.63, 1.38 | 0.7 | 0.79 | 0.64, 0.97 | 0.021 | 0.97 | 0.85, 1.12 | 0.7 | 0.28 | 0.15, 0.54 | <0.001 | <0.001 | <0.001 |
| CTSV | 0.77 | 0.52, 1.14 | 0.2 | 0.7 | 0.57, 0.85 | <0.001 | 0.89 | 0.78, 1.02 | 0.091 | 0.35 | 0.19, 0.63 | <0.001 | <0.001 | <0.001 |
| CDH5 | 1.38 | 1.02, 1.88 | 0.036 | 1.14 | 0.97, 1.33 | 0.1 | 0.95 | 0.85, 1.06 | 0.4 | 1.96 | 1.28, 3.02 | 0.002 | 0.002 | 0.003 |
| SELE | 1.58 | 1.21, 2.07 | <0.001 | 1.28 | 1.12, 1.46 | <0.001 | 0.89 | 0.81, 0.98 | 0.019 | 1.48 | 0.99, 2.22 | 0.055 | <0.001 | <0.001 |
| SCF | 1.7 | 1.12, 2.58 | 0.013 | 0.83 | 0.70, 0.99 | 0.033 | 0.5 | 0.44, 0.56 | <0.001 | 0.65 | 0.40, 1.05 | 0.078 | <0.001 | <0.001 |
| GLO1 | 0.74 | 0.54, 1.00 | 0.048 | 0.77 | 0.67, 0.90 | <0.001 | 1.16 | 1.04, 1.28 | 0.006 | 0.77 | 0.49, 1.22 | 0.3 | <0.001 | <0.001 |
| PRDX1 | 0.69 | 0.48, 1.00 | 0.047 | 0.79 | 0.67, 0.93 | 0.005 | 1.16 | 1.06, 1.27 | <0.001 | 0.86 | 0.53, 1.38 | 0.5 | <0.001 | <0.001 |
| SCF | 1.67 | 1.12, 2.50 | 0.012 | 0.83 | 0.70, 0.98 | 0.028 | 0.5 | 0.44, 0.56 | <0.001 | 0.69 | 0.43, 1.12 | 0.14 | <0.001 | <0.001 |
| FGF-BP1 | 0.68 | 0.48, 0.96 | 0.028 | 1.14 | 1.03, 1.26 | 0.009 | 1.35 | 1.27, 1.44 | <0.001 | 1.25 | 0.96, 1.63 | 0.095 | <0.001 | <0.001 |
| OPG | 1.31 | 0.96, 1.80 | 0.089 | 1.45 | 1.24, 1.69 | <0.001 | 1.2 | 1.07, 1.33 | <0.001 | 2.76 | 1.83, 4.15 | <0.001 | <0.001 | <0.001 |
| CD163 | 1.3 | 0.98, 1.74 | 0.07 | 1.45 | 1.25, 1.67 | <0.001 | 1.15 | 1.05, 1.27 | 0.004 | 2.51 | 1.61, 3.91 | <0.001 | <0.001 | <0.001 |
| GRN | 1.33 | 0.92, 1.92 | 0.13 | 1.41 | 1.17, 1.70 | <0.001 | 1.22 | 1.07, 1.38 | 0.002 | 2.84 | 1.66, 4.88 | <0.001 | <0.001 | <0.001 |
| PLC | 1.32 | 0.97, 1.79 | 0.078 | 1.52 | 1.30, 1.78 | <0.001 | 1.33 | 1.19, 1.48 | <0.001 | 4.05 | 2.53, 6.49 | <0.001 | <0.001 | <0.001 |
| TIMP4 | 1.29 | 0.97, 1.71 | 0.077 | 1.48 | 1.28, 1.70 | <0.001 | 1.15 | 1.05, 1.27 | 0.004 | 2.6 | 1.82, 3.71 | <0.001 | <0.001 | <0.001 |
| TR | 1.08 | 0.82, 1.42 | 0.6 | 1.63 | 1.42, 1.87 | <0.001 | 1.62 | 1.47, 1.79 | <0.001 | 2.45 | 1.65, 3.61 | <0.001 | <0.001 | <0.001 |
| TNFRSF10C | 1.32 | 0.98, 1.77 | 0.067 | 1.22 | 1.05, 1.41 | 0.008 | 0.9 | 0.82, 0.99 | 0.032 | 1.71 | 1.09, 2.67 | 0.019 | <0.001 | <0.001 |
| GDF_15 | 1.06 | 0.85, 1.32 | 0.6 | 1.47 | 1.32, 1.64 | <0.001 | 1.6 | 1.48, 1.74 | <0.001 | 2.52 | 1.92, 3.31 | <0.001 | <0.001 | <0.001 |
| SPON1 | 1.33 | 0.95, 1.86 | 0.1 | 1.57 | 1.34, 1.85 | <0.001 | 1.61 | 1.43, 1.81 | <0.001 | 3.29 | 2.36, 4.58 | <0.001 | <0.001 | <0.001 |
| CXCL16 | 1.26 | 0.91, 1.75 | 0.2 | 1.47 | 1.24, 1.73 | <0.001 | 1.12 | 1.00, 1.26 | 0.044 | 2.51 | 1.57, 4.00 | <0.001 | <0.001 | <0.001 |
| IGFBP | 0.88 | 0.75, 1.04 | 0.13 | 1.12 | 1.03, 1.21 | 0.008 | 1.49 | 1.40, 1.58 | <0.001 | 2.03 | 1.57, 2.63 | <0.001 | <0.001 | <0.001 |
| AP_N | 1.2 | 0.87, 1.66 | 0.3 | 1.22 | 1.04, 1.43 | 0.017 | 1.46 | 1.30, 1.64 | <0.001 | 2.68 | 1.67, 4.28 | <0.001 | <0.001 | <0.001 |
| AXL | 1.26 | 0.91, 1.72 | 0.2 | 1.38 | 1.17, 1.61 | <0.001 | 1.17 | 1.04, 1.30 | 0.006 | 1.91 | 1.20, 3.05 | 0.006 | <0.001 | <0.001 |
| MMP_2 | 1.1 | 0.83, 1.46 | 0.5 | 1.18 | 1.02, 1.36 | 0.022 | 1.34 | 1.22, 1.49 | <0.001 | 2.49 | 1.67, 3.73 | <0.001 | <0.001 | <0.001 |
| TNFSF13B | 1.31 | 1.00, 1.72 | 0.051 | 1.46 | 1.27, 1.67 | <0.001 | 1.16 | 1.06, 1.28 | 0.002 | 1.98 | 1.36, 2.88 | <0.001 | <0.001 | <0.001 |
| U_PAR | 1.18 | 0.88, 1.59 | 0.3 | 1.62 | 1.40, 1.88 | <0.001 | 1.48 | 1.34, 1.65 | <0.001 | 3.99 | 2.67, 5.95 | <0.001 | <0.001 | <0.001 |
| OPN | 0.97 | 0.76, 1.22 | 0.8 | 1.37 | 1.21, 1.55 | <0.001 | 1.51 | 1.39, 1.66 | <0.001 | 2.91 | 1.98, 4.27 | <0.001 | <0.001 | <0.001 |
| CTSD | 1.15 | 0.83, 1.60 | 0.4 | 1.42 | 1.21, 1.67 | <0.001 | 1.34 | 1.20, 1.50 | <0.001 | 2.13 | 1.34, 3.38 | <0.001 | <0.001 | <0.001 |
| PGLYRP1 | 1.23 | 0.94, 1.61 | 0.14 | 1.2 | 1.05, 1.37 | 0.009 | 1.11 | 1.01, 1.22 | 0.034 | 1.87 | 1.27, 2.77 | 0.002 | <0.001 | <0.001 |
| CCL15 | 1.07 | 0.81, 1.42 | 0.6 | 1.34 | 1.17, 1.55 | <0.001 | 1.27 | 1.15, 1.40 | <0.001 | 2.37 | 1.62, 3.49 | <0.001 | <0.001 | <0.001 |
| ST2 | 1.06 | 0.84, 1.34 | 0.6 | 1.58 | 1.41, 1.76 | <0.001 | 1.82 | 1.68, 1.97 | <0.001 | 3.14 | 2.37, 4.15 | <0.001 | <0.001 | <0.001 |
| IGFBP | 1.14 | 0.88, 1.47 | 0.3 | 1.38 | 1.22, 1.57 | <0.001 | 1.58 | 1.44, 1.73 | <0.001 | 2.46 | 1.79, 3.39 | <0.001 | <0.001 | <0.001 |
| IGFBP_2 | 1.12 | 0.87, 1.42 | 0.4 | 1.39 | 1.23, 1.58 | <0.001 | 1.53 | 1.39, 1.67 | <0.001 | 4.33 | 2.65, 7.06 | <0.001 | <0.001 | <0.001 |
| BMP_6 | 0.87 | 0.68, 1.11 | 0.3 | 1.38 | 1.19, 1.59 | <0.001 | 1.72 | 1.55, 1.91 | <0.001 | 2.36 | 1.53, 3.64 | <0.001 | <0.001 | <0.001 |
| ADM | 1.14 | 0.85, 1.54 | 0.4 | 2.11 | 1.75, 2.55 | <0.001 | 2.42 | 2.11, 2.78 | <0.001 | 7.26 | 3.71, 14.2 | <0.001 | <0.001 | <0.001 |
| Placenta growth factor | 1.02 | 0.69, 1.50 | 0.9 | 1.29 | 1.06, 1.57 | 0.009 | 1.34 | 1.17, 1.54 | <0.001 | 2.81 | 1.67, 4.72 | <0.001 | <0.001 | <0.001 |
| ADAM-TS13 | 0.53 | 0.20, 1.40 | 0.2 | 0.28 | 0.18, 0.46 | <0.001 | 0.21 | 0.15, 0.30 | <0.001 | 0.05 | 0.01, 0.14 | <0.001 | <0.001 | <0.001 |
| IL-4RA | 0.96 | 0.63, 1.46 | 0.9 | 1.59 | 1.31, 1.92 | <0.001 | 2.3 | 2.01, 2.63 | <0.001 | 4.19 | 2.76, 6.35 | <0.001 | <0.001 | <0.001 |
| IL6 | 0.91 | 0.74, 1.11 | 0.3 | 1.33 | 1.21, 1.45 | <0.001 | 1.55 | 1.46, 1.65 | <0.001 | 1.85 | 1.50, 2.29 | <0.001 | <0.001 | <0.001 |
| TNFRSF10A | 1.11 | 0.73, 1.69 | 0.6 | 1.79 | 1.45, 2.20 | <0.001 | 2.1 | 1.81, 2.43 | <0.001 | 4.75 | 2.74, 8.25 | <0.001 | <0.001 | <0.001 |
| TNFRSF11A | 1.13 | 0.85, 1.50 | 0.4 | 1.51 | 1.31, 1.74 | <0.001 | 1.59 | 1.44, 1.76 | <0.001 | 2.81 | 1.93, 4.09 | <0.001 | <0.001 | <0.001 |
| PAR_1 | 1.19 | 0.78, 1.82 | 0.4 | 1.36 | 1.10, 1.68 | 0.004 | 1.75 | 1.50, 2.04 | <0.001 | 4.41 | 2.16, 9.00 | <0.001 | <0.001 | <0.001 |
| TRAIL_R2 | 0.96 | 0.68, 1.35 | 0.8 | 1.53 | 1.30, 1.80 | <0.001 | 2.23 | 1.99, 2.50 | <0.001 | 3.23 | 2.42, 4.32 | <0.001 | <0.001 | <0.001 |
| TF | 1.35 | 0.80, 2.27 | 0.3 | 1.57 | 1.20, 2.03 | <0.001 | 1.66 | 1.38, 2.00 | <0.001 | 5.57 | 2.60, 11.9 | <0.001 | <0.001 | <0.001 |
| PDGF subunit B | 0.9 | 0.78, 1.05 | 0.2 | 0.93 | 0.86, 1.00 | 0.048 | 0.95 | 0.90, 1.00 | 0.04 | 0.79 | 0.63, 0.99 | 0.04 | 0.02 | 0.026 |
| IL_27 | 0.97 | 0.62, 1.52 | 0.9 | 2.01 | 1.61, 2.52 | <0.001 | 3.08 | 2.61, 3.63 | <0.001 | 6.23 | 3.23, 12.0 | <0.001 | <0.001 | <0.001 |
| IL_17D | 0.89 | 0.50, 1.58 | 0.7 | 1.42 | 1.10, 1.82 | 0.006 | 1.29 | 1.07, 1.55 | 0.008 | 2.05 | 1.25, 3.34 | 0.004 | 0.003 | 0.004 |
| GAL_9 | 1.19 | 0.66, 2.16 | 0.6 | 2.23 | 1.65, 3.02 | <0.001 | 2.33 | 1.88, 2.88 | <0.001 | 5.52 | 2.22, 13.7 | <0.001 | <0.001 | <0.001 |
| FGF_21 | 0.94 | 0.82, 1.09 | 0.4 | 1.22 | 1.14, 1.30 | <0.001 | 1.33 | 1.27, 1.40 | <0.001 | 1.46 | 1.22, 1.75 | <0.001 | <0.001 | <0.001 |
| FGF_23 | 0.9 | 0.76, 1.07 | 0.2 | 1.36 | 1.28, 1.46 | <0.001 | 1.59 | 1.51, 1.66 | <0.001 | 1.91 | 1.61, 2.25 | <0.001 | <0.001 | <0.001 |
| SPON2 | 1.12 | 0.39, 3.19 | 0.8 | 4.92 | 2.71, 8.93 | <0.001 | 14 | 8.93, 22.0 | <0.001 | 427 | 47.5, 3,841 | <0.001 | <0.001 | <0.001 |
| GH | 0.92 | 0.81, 1.04 | 0.2 | 1.1 | 1.02, 1.17 | 0.007 | 1.31 | 1.25, 1.38 | <0.001 | 1.55 | 1.22, 1.96 | <0.001 | <0.001 | <0.001 |
| FS | 0.8 | 0.55, 1.17 | 0.3 | 1.34 | 1.11, 1.63 | 0.003 | 2.26 | 1.96, 2.60 | <0.001 | 2.49 | 1.41, 4.39 | 0.002 | <0.001 | <0.001 |
| PAPPA | 0.76 | 0.54, 1.05 | 0.1 | 1.24 | 1.06, 1.45 | 0.006 | 1.75 | 1.58, 1.95 | <0.001 | 2.23 | 1.53, 3.25 | <0.001 | <0.001 | <0.001 |
| MERTK | 0.99 | 0.65, 1.50 | 0.9 | 1.24 | 1.01, 1.54 | 0.043 | 1.43 | 1.23, 1.66 | <0.001 | 2.29 | 1.21, 4.32 | 0.011 | <0.001 | <0.001 |
| KIM1 | 1.19 | 0.95, 1.48 | 0.13 | 1.24 | 1.11, 1.38 | <0.001 | 1.39 | 1.28, 1.50 | <0.001 | 1.45 | 1.06, 1.99 | 0.02 | <0.001 | <0.001 |
| THBS2 | 0.62 | 0.27, 1.41 | 0.3 | 3.31 | 2.15, 5.09 | <0.001 | 3.28 | 2.42, 4.46 | <0.001 | 11.6 | 3.15, 42.9 | <0.001 | <0.001 | <0.001 |
| Prolargin | 1.15 | 0.55, 2.41 | 0.7 | 4.13 | 2.82, 6.04 | <0.001 | 9.15 | 6.90, 12.1 | <0.001 | 19.3 | 6.62, 56.5 | <0.001 | <0.001 | <0.001 |
| Heme oxygenase 1 | 0.94 | 0.61, 1.44 | 0.8 | 1.32 | 1.06, 1.66 | 0.015 | 1.92 | 1.63, 2.26 | <0.001 | 3.69 | 1.79, 7.64 | <0.001 | <0.001 | <0.001 |
| Lymphotactin | 0.96 | 0.68, 1.36 | 0.8 | 1.47 | 1.24, 1.75 | <0.001 | 1.52 | 1.35, 1.72 | <0.001 | 2.88 | 1.86, 4.48 | <0.001 | <0.001 | <0.001 |
| PTX3 | 0.79 | 0.56, 1.13 | 0.2 | 1.65 | 1.38, 1.98 | <0.001 | 2.56 | 2.24, 2.92 | <0.001 | 3.69 | 2.19, 6.19 | <0.001 | <0.001 | <0.001 |
| PSGL_1 | 0.81 | 0.37, 1.77 | 0.6 | 0.58 | 0.40, 0.86 | 0.007 | 0.59 | 0.45, 0.78 | <0.001 | 0.21 | 0.07, 0.62 | 0.005 | <0.001 | <0.001 |
| CCL3 | 0.73 | 0.52, 1.02 | 0.067 | 1.35 | 1.15, 1.58 | <0.001 | 1.62 | 1.45, 1.81 | <0.001 | 2.21 | 1.50, 3.27 | <0.001 | <0.001 | <0.001 |
| DCN | 0.86 | 0.48, 1.55 | 0.6 | 1.98 | 1.49, 2.64 | <0.001 | 2.61 | 2.13, 3.20 | <0.001 | 8.35 | 4.24, 16.4 | <0.001 | <0.001 | <0.001 |
| AGRP | 0.81 | 0.56, 1.18 | 0.3 | 1.47 | 1.22, 1.77 | <0.001 | 2.01 | 1.76, 2.29 | <0.001 | 3.35 | 2.00, 5.63 | <0.001 | <0.001 | <0.001 |
| PD_L2 | 1.06 | 0.64, 1.75 | 0.8 | 1.46 | 1.13, 1.88 | 0.003 | 2.29 | 1.91, 2.74 | <0.001 | 5.22 | 2.63, 10.4 | <0.001 | <0.001 | <0.001 |
| hOSCAR | 1.03 | 0.51, 2.10 | 0.9 | 2.3 | 1.50, 3.52 | <0.001 | 1.51 | 1.13, 2.01 | 0.005 | 15.5 | 3.98, 60.5 | <0.001 | <0.001 | <0.001 |
| CD4 | 0.95 | 0.56, 1.60 | 0.8 | 1.76 | 1.36, 2.27 | <0.001 | 2.27 | 1.89, 2.73 | <0.001 | 4.55 | 2.37, 8.72 | <0.001 | <0.001 | <0.001 |
| CLEC4G | 0.88 | 0.54, 1.43 | 0.6 | 1.57 | 1.23, 2.00 | <0.001 | 1.71 | 1.43, 2.03 | <0.001 | 2.33 | 1.13, 4.82 | 0.022 | <0.001 | <0.001 |
| IL6 | 0.91 | 0.74, 1.12 | 0.4 | 1.33 | 1.21, 1.45 | <0.001 | 1.54 | 1.44, 1.64 | <0.001 | 1.83 | 1.49, 2.25 | <0.001 | <0.001 | <0.001 |
| DPP10 | 0.9 | 0.60, 1.34 | 0.6 | 1.35 | 1.14, 1.60 | <0.001 | 1.67 | 1.49, 1.88 | <0.001 | 1.77 | 1.15, 2.73 | 0.009 | <0.001 | <0.001 |
| CDSN | 1.15 | 0.83, 1.61 | 0.4 | 1.24 | 1.05, 1.46 | 0.013 | 1.51 | 1.34, 1.70 | <0.001 | 2.57 | 1.60, 4.11 | <0.001 | <0.001 | <0.001 |
| LILRB4 | 0.91 | 0.65, 1.26 | 0.6 | 1.74 | 1.49, 2.04 | <0.001 | 1.83 | 1.64, 2.05 | <0.001 | 3.63 | 2.38, 5.51 | <0.001 | <0.001 | <0.001 |
| KRT19 | 1.14 | 0.88, 1.48 | 0.3 | 1.25 | 1.10, 1.42 | <0.001 | 1.37 | 1.26, 1.50 | <0.001 | 2.13 | 1.61, 2.81 | <0.001 | <0.001 | <0.001 |
| ITM2A | 1.01 | 0.73, 1.41 | 0.9 | 1.21 | 1.02, 1.43 | 0.029 | 1.46 | 1.29, 1.65 | <0.001 | 2.5 | 1.50, 4.16 | <0.001 | <0.001 | <0.001 |
| MILR1 | 0.93 | 0.63, 1.36 | 0.7 | 1.78 | 1.49, 2.12 | <0.001 | 1.8 | 1.58, 2.04 | <0.001 | 3.61 | 2.23, 5.85 | <0.001 | <0.001 | <0.001 |
| CD28 | 1.42 | 0.98, 2.06 | 0.067 | 1.33 | 1.09, 1.62 | 0.005 | 1.17 | 1.00, 1.36 | 0.044 | 1.65 | 1.01, 2.68 | 0.045 | 0.008 | 0.011 |
| CKAP4 | 1.01 | 0.70, 1.44 | 0.9 | 1.72 | 1.46, 2.03 | <0.001 | 1.91 | 1.70, 2.16 | <0.001 | 3.13 | 2.16, 4.54 | <0.001 | <0.001 | <0.001 |
| CLEC4D | 0.92 | 0.69, 1.23 | 0.6 | 1.29 | 1.13, 1.48 | <0.001 | 1.36 | 1.24, 1.50 | <0.001 | 1.54 | 1.05, 2.27 | 0.028 | <0.001 | <0.001 |
| TREM1 | 0.89 | 0.61, 1.29 | 0.5 | 1.41 | 1.17, 1.70 | <0.001 | 2.04 | 1.78, 2.34 | <0.001 | 4.08 | 2.32, 7.16 | <0.001 | <0.001 | <0.001 |
| CXADR | 0.97 | 0.70, 1.36 | 0.9 | 1.41 | 1.20, 1.64 | <0.001 | 1.96 | 1.76, 2.19 | <0.001 | 3.15 | 2.15, 4.60 | <0.001 | <0.001 | <0.001 |
| IL10 | 1.16 | 0.89, 1.51 | 0.3 | 1.38 | 1.23, 1.55 | <0.001 | 1.32 | 1.21, 1.45 | <0.001 | 1.43 | 1.05, 1.94 | 0.024 | <0.001 | <0.001 |
| NCR1 | 1.22 | 0.88, 1.71 | 0.2 | 1.4 | 1.19, 1.66 | <0.001 | 1.16 | 1.03, 1.30 | 0.017 | 1.73 | 1.07, 2.79 | 0.026 | <0.001 | <0.001 |
| AREG | 0.97 | 0.69, 1.35 | 0.8 | 1.56 | 1.34, 1.82 | <0.001 | 2.03 | 1.82, 2.27 | <0.001 | 2.96 | 2.05, 4.28 | <0.001 | <0.001 | <0.001 |
| CLEC7A | 1.01 | 0.77, 1.33 | 0.9 | 1.27 | 1.11, 1.46 | <0.001 | 1.39 | 1.25, 1.53 | <0.001 | 2.38 | 1.60, 3.54 | <0.001 | <0.001 | <0.001 |
| CLEC6A | 0.95 | 0.63, 1.41 | 0.8 | 1.54 | 1.28, 1.85 | <0.001 | 1.39 | 1.22, 1.59 | <0.001 | 1.82 | 1.09, 3.04 | 0.021 | <0.001 | <0.001 |
| IL12RB1 | 1.18 | 0.73, 1.89 | 0.5 | 1.49 | 1.18, 1.87 | <0.001 | 1.53 | 1.30, 1.80 | <0.001 | 2.04 | 1.11, 3.75 | 0.022 | <0.001 | <0.001 |
| ITGA11 | 1.36 | 0.88, 2.08 | 0.2 | 0.71 | 0.56, 0.89 | 0.003 | 0.63 | 0.54, 0.74 | <0.001 | 0.37 | 0.19, 0.73 | 0.004 | <0.001 | <0.001 |
| LAG3 | 0.92 | 0.62, 1.38 | 0.7 | 1.38 | 1.15, 1.66 | <0.001 | 1.46 | 1.28, 1.67 | <0.001 | 2.09 | 1.34, 3.26 | <0.001 | <0.001 | <0.001 |
| BTN3A2 | 1.11 | 0.75, 1.65 | 0.6 | 1.41 | 1.16, 1.72 | <0.001 | 1.52 | 1.33, 1.75 | <0.001 | 3.01 | 1.77, 5.11 | <0.001 | <0.001 | <0.001 |
| VEGFA | 0.84 | 0.55, 1.28 | 0.4 | 1.43 | 1.16, 1.75 | <0.001 | 1.85 | 1.60, 2.14 | <0.001 | 3.06 | 1.80, 5.21 | <0.001 | <0.001 | <0.001 |
| TNFSF13 | 1.14 | 0.72, 1.80 | 0.6 | 2.15 | 1.69, 2.74 | <0.001 | 2.73 | 2.29, 3.26 | <0.001 | 8.26 | 4.01, 17.0 | <0.001 | <0.001 | <0.001 |
| TNFRSF6B | 1.07 | 0.82, 1.39 | 0.6 | 1.54 | 1.36, 1.74 | <0.001 | 1.68 | 1.54, 1.84 | <0.001 | 3.35 | 2.45, 4.60 | <0.001 | <0.001 | <0.001 |
| IL6 | 0.91 | 0.75, 1.12 | 0.4 | 1.32 | 1.21, 1.43 | <0.001 | 1.53 | 1.44, 1.63 | <0.001 | 1.84 | 1.50, 2.26 | <0.001 | <0.001 | <0.001 |
| CD48 | 1.15 | 0.62, 2.12 | 0.7 | 1.54 | 1.13, 2.09 | 0.006 | 1.42 | 1.14, 1.77 | 0.002 | 4.15 | 1.90, 9.07 | <0.001 | <0.001 | <0.001 |
| LY9 | 1.04 | 0.63, 1.72 | 0.9 | 1.35 | 1.05, 1.74 | 0.018 | 1.67 | 1.40, 1.99 | <0.001 | 3.43 | 1.74, 6.76 | <0.001 | <0.001 | <0.001 |
| IFN-gamma-R1 | 1.14 | 0.66, 1.96 | 0.6 | 2.27 | 1.75, 2.96 | <0.001 | 2.56 | 2.12, 3.10 | <0.001 | 6.86 | 3.72, 12.7 | <0.001 | <0.001 | <0.001 |
| TRAIL | 0.64 | 0.38, 1.06 | 0.081 | 0.71 | 0.55, 0.92 | 0.009 | 0.42 | 0.35, 0.51 | <0.001 | 0.24 | 0.13, 0.45 | <0.001 | <0.001 | <0.001 |
| hK11 | 0.96 | 0.66, 1.41 | 0.9 | 1.23 | 1.02, 1.48 | 0.032 | 1.87 | 1.64, 2.13 | <0.001 | 3.49 | 2.15, 5.67 | <0.001 | <0.001 | <0.001 |
| TFPI-2 | 0.86 | 0.62, 1.20 | 0.4 | 1.56 | 1.35, 1.80 | <0.001 | 1.86 | 1.68, 2.06 | <0.001 | 1.76 | 1.19, 2.60 | 0.005 | <0.001 | <0.001 |
| VEGFR-2 | 1.16 | 0.56, 2.40 | 0.7 | 0.5 | 0.35, 0.70 | <0.001 | 0.49 | 0.38, 0.63 | <0.001 | 0.17 | 0.07, 0.39 | <0.001 | <0.001 | <0.001 |
| TGF-alpha | 0.79 | 0.49, 1.25 | 0.3 | 1.81 | 1.45, 2.26 | <0.001 | 2.6 | 2.22, 3.05 | <0.001 | 4.95 | 2.87, 8.55 | <0.001 | <0.001 | <0.001 |
| EPHA2 | 1.4 | 0.93, 2.11 | 0.11 | 1.66 | 1.36, 2.03 | <0.001 | 1.93 | 1.67, 2.23 | <0.001 | 4.68 | 2.80, 7.84 | <0.001 | <0.001 | <0.001 |
| ITGB5 | 1.04 | 0.59, 1.85 | 0.9 | 1.4 | 1.05, 1.86 | 0.021 | 1.82 | 1.49, 2.23 | <0.001 | 3.4 | 1.64, 7.04 | <0.001 | <0.001 | <0.001 |
| GPNMB | 1.19 | 0.49, 2.91 | 0.7 | 2.06 | 1.30, 3.26 | 0.002 | 5.36 | 3.82, 7.54 | <0.001 | 12 | 2.89, 49.5 | <0.001 | <0.001 | <0.001 |
| CAIX | 0.88 | 0.67, 1.16 | 0.4 | 1.29 | 1.13, 1.47 | <0.001 | 1.54 | 1.41, 1.69 | <0.001 | 1.94 | 1.37, 2.74 | <0.001 | <0.001 | <0.001 |
| CD27 | 1.48 | 1.00, 2.20 | 0.051 | 1.27 | 1.04, 1.55 | 0.018 | 1.41 | 1.22, 1.62 | <0.001 | 3.91 | 2.21, 6.92 | <0.001 | <0.001 | <0.001 |
| ADAM 8 | 1.37 | 0.84, 2.23 | 0.2 | 1.37 | 1.08, 1.75 | 0.011 | 1.32 | 1.11, 1.56 | 0.002 | 3.08 | 1.54, 6.14 | <0.001 | <0.001 | <0.001 |
| 5'-NT | 1.03 | 0.78, 1.36 | 0.9 | 1.43 | 1.25, 1.64 | <0.001 | 1.73 | 1.57, 1.91 | <0.001 | 2.35 | 1.55, 3.56 | <0.001 | <0.001 | <0.001 |
| DLL1 | 1.45 | 0.86, 2.43 | 0.2 | 1.86 | 1.43, 2.42 | <0.001 | 2.11 | 1.75, 2.54 | <0.001 | 5.93 | 2.55, 13.8 | <0.001 | <0.001 | <0.001 |
| VIM | 0.76 | 0.55, 1.05 | 0.1 | 1.44 | 1.23, 1.68 | <0.001 | 1.95 | 1.74, 2.18 | <0.001 | 3.07 | 2.00, 4.72 | <0.001 | <0.001 | <0.001 |
| TNFRSF19 | 1.12 | 0.85, 1.48 | 0.4 | 1.2 | 1.05, 1.38 | 0.009 | 1.46 | 1.33, 1.61 | <0.001 | 2.65 | 1.88, 3.75 | <0.001 | <0.001 | <0.001 |
| TCL1A | 1.06 | 0.90, 1.25 | 0.5 | 0.88 | 0.81, 0.96 | 0.005 | 0.88 | 0.83, 0.94 | <0.001 | 0.74 | 0.57, 0.96 | 0.025 | <0.001 | <0.001 |
| WISP-1 | 1.13 | 0.79, 1.61 | 0.5 | 2.04 | 1.72, 2.42 | <0.001 | 2.75 | 2.43, 3.11 | <0.001 | 5.85 | 3.76, 9.10 | <0.001 | <0.001 | <0.001 |
| CXL17 | 0.82 | 0.59, 1.14 | 0.2 | 1.22 | 1.03, 1.45 | 0.019 | 1.46 | 1.30, 1.65 | <0.001 | 2.65 | 1.69, 4.14 | <0.001 | <0.001 | <0.001 |
| PPY | 1.02 | 0.85, 1.21 | 0.8 | 1.13 | 1.03, 1.23 | 0.01 | 1.26 | 1.18, 1.35 | <0.001 | 1.4 | 1.04, 1.87 | 0.025 | <0.001 | <0.001 |
| AREG | 0.98 | 0.68, 1.42 | 0.9 | 1.59 | 1.35, 1.89 | <0.001 | 2.09 | 1.86, 2.36 | <0.001 | 3.3 | 2.24, 4.84 | <0.001 | <0.001 | <0.001 |
| WFDC2 | 1.18 | 0.74, 1.87 | 0.5 | 1.62 | 1.27, 2.05 | <0.001 | 2.73 | 2.28, 3.26 | <0.001 | 8.17 | 3.81, 17.5 | <0.001 | <0.001 | <0.001 |
| CXCL13 | 0.97 | 0.73, 1.28 | 0.8 | 1.34 | 1.18, 1.52 | <0.001 | 1.42 | 1.30, 1.55 | <0.001 | 2.05 | 1.50, 2.80 | <0.001 | <0.001 | <0.001 |
| CD70 | 1.36 | 0.95, 1.96 | 0.093 | 1.66 | 1.39, 1.99 | <0.001 | 1.19 | 1.04, 1.36 | 0.011 | 2.18 | 1.41, 3.37 | <0.001 | <0.001 | <0.001 |
| RSPO3 | 1.07 | 0.86, 1.35 | 0.5 | 1.33 | 1.20, 1.48 | <0.001 | 1.41 | 1.31, 1.52 | <0.001 | 1.95 | 1.56, 2.45 | <0.001 | <0.001 | <0.001 |
| FCRLB | 0.76 | 0.54, 1.07 | 0.12 | 1.38 | 1.21, 1.57 | <0.001 | 1.57 | 1.43, 1.73 | <0.001 | 2.1 | 1.65, 2.69 | <0.001 | <0.001 | <0.001 |
| TLT_2 | 1.52 | 1.15, 2.02 | 0.003 | 1.06 | 0.91, 1.22 | 0.5 | 0.74 | 0.67, 0.82 | <0.001 | 1.64 | 1.08, 2.47 | 0.02 | <0.001 | <0.001 |
| T_PA | 0.8 | 0.66, 0.96 | 0.02 | 1.07 | 0.99, 1.17 | 0.1 | 1.14 | 1.07, 1.20 | <0.001 | 1.35 | 1.08, 1.68 | 0.008 | <0.001 | <0.001 |
| NT_pro_BNP | 0.71 | 0.58, 0.86 | <0.001 | 0.92 | 0.85, 1.01 | 0.077 | 1.81 | 1.70, 1.92 | <0.001 | 2.27 | 1.84, 2.80 | <0.001 | <0.001 | <0.001 |
| WIF-1 | 0.61 | 0.40, 0.93 | 0.023 | 1.21 | 0.98, 1.49 | 0.074 | 2.41 | 2.08, 2.80 | <0.001 | 3.19 | 1.79, 5.69 | <0.001 | <0.001 | <0.001 |
| EPHB4 | 1.61 | 1.15, 2.26 | 0.006 | 1.44 | 1.20, 1.71 | <0.001 | 1.11 | 0.98, 1.27 | 0.1 | 3.11 | 2.07, 4.65 | <0.001 | <0.001 | <0.001 |
| ALCAM | 1.56 | 1.10, 2.22 | 0.012 | 1.4 | 1.17, 1.67 | <0.001 | 1 | 0.88, 1.12 | 0.9 | 2.46 | 1.53, 3.95 | <0.001 | <0.001 | <0.001 |
| MCP_1 | 1.48 | 1.12, 1.94 | 0.005 | 1.2 | 1.03, 1.41 | 0.021 | 0.96 | 0.85, 1.07 | 0.4 | 1.7 | 1.21, 2.39 | 0.002 | <0.001 | <0.001 |
| IL_18BP | 1.46 | 1.10, 1.94 | 0.008 | 1.4 | 1.22, 1.62 | <0.001 | 1 | 0.91, 1.10 | 0.9 | 2.86 | 1.91, 4.28 | <0.001 | <0.001 | <0.001 |
| pdgf_subunit_a | 0.61 | 0.48, 0.79 | <0.001 | 0.81 | 0.72, 0.90 | <0.001 | 1.01 | 0.94, 1.08 | 0.8 | 0.57 | 0.39, 0.84 | 0.004 | <0.001 | <0.001 |
| tnfrsf14 | 1.33 | 1.02, 1.72 | 0.033 | 1.26 | 1.10, 1.43 | <0.001 | 1.15 | 1.05, 1.26 | 0.003 | 2.58 | 1.84, 3.62 | <0.001 | <0.001 | <0.001 |
| tnf_r2 | 1.36 | 1.07, 1.73 | 0.013 | 1.43 | 1.27, 1.62 | <0.001 | 1.19 | 1.09, 1.30 | <0.001 | 2.66 | 1.96, 3.61 | <0.001 | <0.001 | <0.001 |
| il2_ra | 1.37 | 1.06, 1.77 | 0.017 | 1.33 | 1.16, 1.51 | <0.001 | 1.11 | 1.01, 1.22 | 0.028 | 2.39 | 1.72, 3.31 | <0.001 | <0.001 | <0.001 |
| tff3 | 1.27 | 1.01, 1.60 | 0.045 | 1.35 | 1.21, 1.52 | <0.001 | 1.33 | 1.22, 1.44 | <0.001 | 2.49 | 1.87, 3.30 | <0.001 | <0.001 | <0.001 |
| gal_3 | 1.48 | 1.08, 2.03 | 0.014 | 1.2 | 1.02, 1.40 | 0.027 | 0.81 | 0.72, 0.90 | <0.001 | 1.98 | 1.27, 3.08 | 0.003 | <0.001 | <0.001 |
| ltbr | 1.43 | 1.07, 1.89 | 0.015 | 1.47 | 1.28, 1.70 | <0.001 | 1.22 | 1.10, 1.35 | <0.001 | 3.37 | 2.33, 4.88 | <0.001 | <0.001 | <0.001 |
| notch_3 | 1.52 | 1.14, 2.04 | 0.005 | 1.52 | 1.31, 1.76 | <0.001 | 1.3 | 1.17, 1.45 | <0.001 | 2.91 | 2.06, 4.11 | <0.001 | <0.001 | <0.001 |
| cntn1 | 1.54 | 1.12, 2.11 | 0.008 | 1.18 | 1.00, 1.39 | 0.047 | 0.81 | 0.73, 0.91 | <0.001 | 1.82 | 1.16, 2.87 | 0.01 | <0.001 | <0.001 |
| fabp4 | 1.27 | 1.07, 1.51 | 0.006 | 1.51 | 1.38, 1.64 | <0.001 | 1.32 | 1.24, 1.40 | <0.001 | 1.88 | 1.48, 2.39 | <0.001 | <0.001 | <0.001 |
| dlk_1 | 1.46 | 1.17, 1.82 | <0.001 | 1.18 | 1.06, 1.32 | 0.003 | 0.88 | 0.81, 0.95 | <0.001 | 1.88 | 1.36, 2.58 | <0.001 | <0.001 | <0.001 |
| il_1rt1 | 1.42 | 1.03, 1.95 | 0.033 | 1.3 | 1.11, 1.53 | <0.001 | 1.26 | 1.13, 1.41 | <0.001 | 3.14 | 2.05, 4.80 | <0.001 | <0.001 | <0.001 |
| mb | 1.24 | 1.01, 1.52 | 0.036 | 1.16 | 1.04, 1.29 | 0.005 | 1.09 | 1.01, 1.17 | 0.022 | 1.7 | 1.30, 2.21 | <0.001 | <0.001 | <0.001 |
| chi3l1 | 1.3 | 1.07, 1.56 | 0.007 | 1.29 | 1.17, 1.42 | <0.001 | 1.11 | 1.04, 1.18 | 0.002 | 2.26 | 1.66, 3.09 | <0.001 | <0.001 | <0.001 |
| col1a1 | 1.48 | 1.06, 2.06 | 0.021 | 1.43 | 1.21, 1.69 | <0.001 | 1.16 | 1.03, 1.30 | 0.013 | 3.59 | 2.16, 5.95 | <0.001 | <0.001 | <0.001 |
| ctsz | 1.42 | 1.04, 1.96 | 0.03 | 1.33 | 1.13, 1.56 | <0.001 | 0.86 | 0.78, 0.96 | 0.005 | 2.19 | 1.36, 3.54 | <0.001 | <0.001 | <0.001 |
| vwf | 0.78 | 0.65, 0.94 | 0.009 | 1.12 | 1.02, 1.22 | 0.013 | 1.09 | 1.03, 1.16 | 0.005 | 1.35 | 1.05, 1.73 | 0.02 | <0.001 | <0.001 |
| BNP | 0.74 | 0.65, 0.84 | <0.001 | 0.9 | 0.84, 0.96 | <0.001 | 1.58 | 1.50, 1.66 | <0.001 | 1.79 | 1.45, 2.21 | <0.001 | <0.001 | <0.001 |
| CTSL1 | 0.56 | 0.36, 0.88 | 0.011 | 1.95 | 1.56, 2.43 | <0.001 | 2.85 | 2.43, 3.35 | <0.001 | 5.33 | 2.96, 9.58 | <0.001 | <0.001 | <0.001 |
| LEP | 1.55 | 1.28, 1.88 | <0.001 | 1.33 | 1.21, 1.46 | <0.001 | 0.77 | 0.72, 0.81 | <0.001 | 0.75 | 0.60, 0.94 | 0.013 | <0.001 | <0.001 |
| SYND1 | 0.61 | 0.44, 0.84 | 0.003 | 1.3 | 1.11, 1.52 | <0.001 | 2.21 | 1.97, 2.47 | <0.001 | 2.19 | 1.39, 3.43 | <0.001 | <0.001 | <0.001 |
| TGFR-2 | 1.76 | 1.17, 2.65 | 0.006 | 1.74 | 1.42, 2.14 | <0.001 | 1.47 | 1.27, 1.70 | <0.001 | 4.31 | 2.39, 7.76 | <0.001 | <0.001 | <0.001 |
| HGF | 0.71 | 0.52, 0.98 | 0.035 | 1.36 | 1.20, 1.53 | <0.001 | 1.7 | 1.56, 1.85 | <0.001 | 1.85 | 1.36, 2.50 | <0.001 | <0.001 | <0.001 |
| MK | 0.77 | 0.60, 0.98 | 0.037 | 1.19 | 1.07, 1.33 | <0.001 | 1.42 | 1.32, 1.53 | <0.001 | 1.5 | 1.12, 2.01 | 0.007 | <0.001 | <0.001 |
| ESM-1 | 0.64 | 0.46, 0.90 | 0.009 | 1.47 | 1.25, 1.73 | <0.001 | 2.3 | 2.04, 2.59 | <0.001 | 3.21 | 1.97, 5.25 | <0.001 | <0.001 | <0.001 |
| MUC-16 | 0.79 | 0.67, 0.92 | 0.002 | 1.23 | 1.15, 1.32 | <0.001 | 1.52 | 1.44, 1.60 | <0.001 | 2.48 | 1.92, 3.20 | <0.001 | <0.001 | <0.001 |

**Table 3S: Association of identified phenotype with composite outcome in the Nancy-HF after adjustment for clinical variable and treatment.**

| Phenotype | N events /N | Nancy-HF | |  |
| --- | --- | --- | --- | --- |
|  |  | HR (95% CI) | p-value |  |
| Model 2 + treatment |  |  |  |  |
| Pulm-tissue congestion with dilated LV |  | Ref |  |  |
| Pulm Tissue congestion with HFpEF |  | 1.14 (0.66 - 1.96) | 0.64 |  |
| Pulm tissue and Syst tissue congestion with AF |  | 1.70 (1.10 - 2.64) | 0.018 |  |
| Pulm IV congestion with dilated LA & LV |  | 1.16 (0.76 - 1.77) | 0.48 |  |
| Global congestion |  | 2.39 (1.57 - 3.64) | <0.0001 |  |

Reference: Pulm-tissue congestion with dilated LV

Model 1: adjusted for age, sex, BMI, SBP, LVEF, eGFR.

Model 2: Model M1 + NT-proBNP z score

The outcome was a composite of re-hospitalization for HF or death in the derivation cohort (Nancy-HF) and a composite of death and hospitalization in validation cohort (BIOSTAT-CHF).
